# Identification of isoform switching events linked with esophageal adenocarcinoma patient survival informs novel prognostic and therapeutic targets

**DOI:** 10.1038/s41419-026-08542-2

**Published:** 2026-03-11

**Authors:** Yun Zhang, David A. Ntsiful, Rachel Israel, Bryce Vandenburg, Shari Barnett, Jean-Jack Riethoven, Jennifer L. Clarke, Kiran H. Lagisetty, Jules Lin, Rishindra M. Reddy, Andrew C. Chang, David D. Odell, Analisa DiFeo, Maureen A. Sartor, Laura A. Kresty

**Affiliations:** 1 Section of Thoracic Surgery, Department of Surgery, University of Michigan, Ann Arbor, MI USA; 2 Nebraska Center for Biotechnology, University of Nebraska – Lincoln, Lincoln, NE USA; 3 Nebraska Center for Integrated Biomolecular Communication, University of Nebraska, Lincoln, NE USA; 4 Department of Food Science Technology, University of Nebraska, Lincoln, NE USA; 5 Department of Pathology, University of Michigan, Ann Arbor, MI USA; 6 Department of Computational Medicine and Bioinformatics, University of Michigan, Ann Arbor, MI USA

**Keywords:** Oesophageal cancer, Transcriptomics

## Abstract

Esophageal adenocarcinoma (EAC), the dominant subtype of esophageal cancer in developed countries, is a growing health problem, characterized by poor patient prognosis and dismal survival due to ineffective screening tools and a lack of efficacious options targeting the interception or treatment of EAC. Despite molecular advances, molecular targeting of EAC remains elusive, suggesting the need for identifying alternative targets with improved prognostic and therapeutic value. Herein, we performed RNA-sequencing analysis in EAC and Barrett’s Esophagus (BE) precursor lesions to identify isoform switching events significantly linked with all-cause and cancer-specific mortality. Patients were stratified based on histopathology alone or in combination with *TP53* mutation status, the most commonly mutated gene in EAC. To gain mechanistic insight, we performed isoform-specific siRNA knockdown of two isoforms, *TTLL12* and *HM13*, both linked to patient survival, and investigated mechanisms associated with isoform dysregulation and whether targeting specific isoforms in EAC acts synergistically to improve therapeutic potential. Isoform-specific knockdown of *TTLL12* and *HM13* significantly decreased the viability of two EAC cell lines, sensitized EAC cell lines to standard-of-care chemotherapy agents (paclitaxel and carboplatin) with synergy, and inhibited EAC cell migratory potential. Knockdown of the *TTLL12* isoform led to activation of chaperone-mediated autophagy, which, in turn, decreased expression of CHK1 and TP53; whereas knockdown of the *HM13* isoform activated the unfolded protein response and induced endoplasmic reticulum stress-induced autophagy and apoptosis. In addition, *HM13* isoform knockdown increased the response to an anti-PD-L1 agent, avelumab, in EAC cells, suggesting a role for isoform switching in immunosuppression. Taken together, study results suggest that isoform switching may provide novel insight for the identification of prognostic markers and inform new potential therapeutic targets for EAC treatment or prevention.

## Introduction

Esophageal adenocarcinoma (EAC), the dominant subtype of esophageal cancer in developed countries such as the United States (US), represents a growing health problem characterized by rising incidence since the 1970s and continued poor prognosis [1]. It is the 6th leading cause of cancer-related mortality worldwide and the 7th among males in the US, with a five-year survival rate of 22% [2, 3]. Ineffective screening tools and late-stage diagnosis, coupled with the lack of efficacious treatment regimens, contribute to the abysmal survival rate of EAC. The only known precursor lesion to EAC is Barrett’s Esophagus (BE), a premalignant condition where the lining of the esophagus changes from a squamous epithelium to a columnar epithelium. BE with metaplasia or BE with low-grade dysplasia (LGD) has a lower risk of progressing to EAC, whereas BE with high-grade dysplasia (HGD) is considered to be at high risk of progression [4]. Although endoscopic surveillance is recommended for patients with BE, only 7.3% of EAC patients have a prior BE diagnosis [5], reinforcing that better risk-prediction models are needed. For patients diagnosed with EAC, the most common standard-of-care treatment is neoadjuvant chemoradiotherapy, followed by esophagectomy for surgery-eligible patients. Concurrent prescription of paclitaxel and carboplatin is commonly utilized to treat EAC [6]. However, only 23.1% of EAC patients achieve a pathological complete response with this treatment regimen, and even these patients are at risk for recurrence [7]. EAC is a heterogeneous cancer dominated by high mutation rates, increased copy number alterations, and large-scale genomic alterations compared to other cancer types [8, 9]. Although it is a cancer with a high mutational burden, it lacks dominant mutations that can be clinically targeted. Over 80% of EAC patients carry a *TP53* mutation, yet mutation frequencies of multiple other genes are all lower than 40% [10]. For these reasons, the identification of alternative targets in BE and EAC is urgently needed for improving patient outcomes.

We previously performed RNA sequencing (RNA-seq) of 57 esophageal tissue samples derived from treatment-naïve HGD and EAC patients undergoing esophagectomy and investigated isoform switching (IS) events linked with EAC progression [11]. IS is defined as the change of dominant gene isoforms between two conditions. We identified 75 genes that are isoform switched between BE with LGD (BE.LGD) and BE with HGD (BE.HGD). The inclusion of *TP53* mutation status in the analysis further increased the number to 135. Importantly, over 40% of identified isoform-switched genes are not differentially expressed at the gene level [11]. Herein, we extended our investigation to assess whether IS events are significantly linked to patient prognosis based on cancer-specific and all-cause mortality, followed by mechanistic studies targeting two isoforms linked to patient survival (*TTLL12* and *HM13* isoforms). Results show that siRNA knockdown of *TTLL12* and *HM13* isoforms significantly sensitized EAC cells to standard-of-care chemotherapeutic agents while having minimal effect on normal esophageal epithelial cells (Het-1A). Mechanisms implicated include activation of chaperone-mediated autophagy (CMA) for the *TTLL12* isoform and unfolded protein response (UPR) and immunosuppression for the *HM13* isoform. Moving forward, specific IS events may inform new potential therapeutic targets for EAC treatment and be developed as prognostic markers.

## Results

### Isoform switching analysis reveals isoforms significantly linked with patient survival

**Fig. 1:**
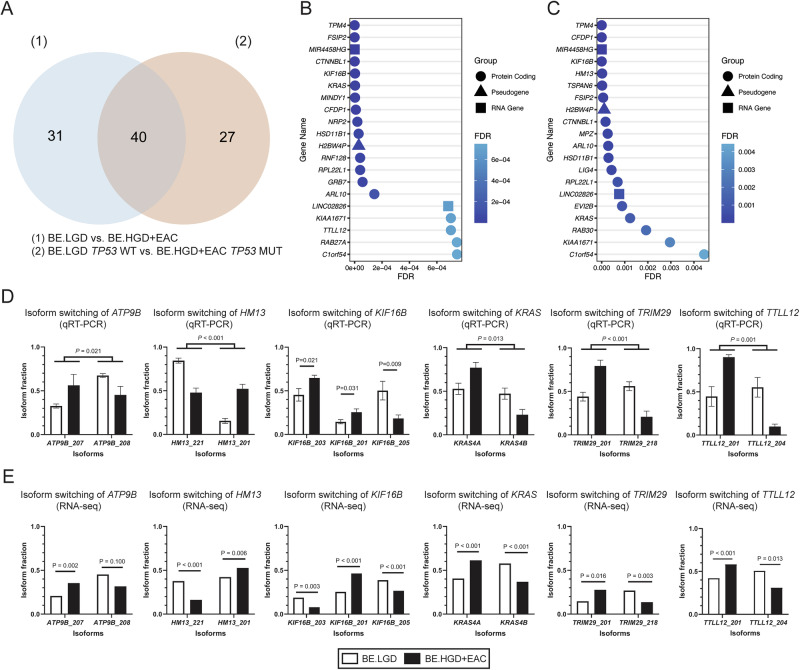
Isoform switching events between BE.LGD versus (vs.) BE.HGD + EAC tissue samples. **A** Venn diagram showing common and unique isoform-switched genes between BE.LGD vs. BE.HGD + EAC (*n* = 71) and BE.LGD *TP53* WT vs. BE.HGD + EAC *TP53* MUT (*n* = 67). Top 20 most significant isoform-switched genes in **B** BE.LGD vs. BE.HGD + EAC and **C** BE.LGD *TP53* WT vs. BE.HGD + EAC *TP53* MUT. FDR, false discovery rate. Isoform fractions determined using **D** qRT-PCR and **E** RNA-seq data. Significant differences in isoform fractions were determined by one-tailed two-sample *t*-tests. Bar plots represent the mean isoform fraction, and error bars indicate the standard error of the mean.

To identify isoforms linked with EAC patient survival, IS analysis was performed utilizing patient samples derived from BE.LGD compared to BE.HGD + EAC, alone or in combination with *TP53* mutation status. As shown in Fig. 1A, 71 genes were isoform-switched compared to BE.LGD and BE.HGD + EAC patient samples. With the inclusion of *TP53* mutation status, a total of 67 genes were isoform-switched. The Venn diagram revealed that 40 isoform-switched genes were shared between the two comparisons (Figs. 1A and S1).

The top 20 most significant isoform-switched genes comparing BE.LGD and BE.HGD + EAC are shown in Fig. 1B. Eighty-five percent (17/20) of the top isoform-switched genes were protein-coding genes, including *TPM4*, *KRAS*, and *TTLL12*. Two RNA genes, *MIR4458HG* and *LINC02826*, and a pseudogene, *H2BW4P*, also showed significant IS. With the inclusion of *TP53* mutation status, 85% (17/20) of the top isoform-switched genes remain protein-coding genes, including *TPM4*, *HM13*, and *KRAS* (Fig. 1C). Among the top 20 genes reported in the two comparisons, 70% (14/20) were shared. Isoform-specific primers were designed to validate specific IS events using qRT-PCR (Fig. 1D), as shown for *ATP9B*, *HM13*, *KIF16B*, *KRAS*, *TRIM29*, and *TTLL12*, with parallel results from the RNA-seq data (Fig. 1E).

**Fig. 2:**
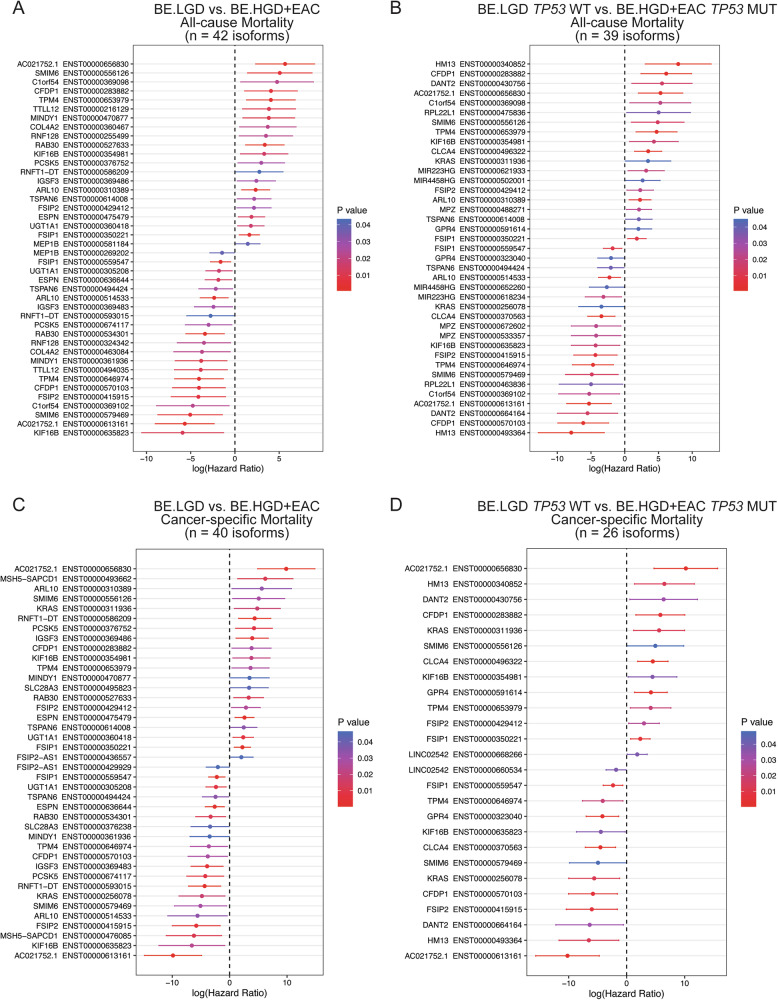
Identified isoforms significantly linked with all-cause or cancer-specific patient mortality based on *TP53* mutation status and pathologic progression to EAC. Isoforms significantly linked with all-cause patient mortality in **A** BE.LGD vs. BE.HGD + EAC and **B** BE.LGD *TP53* WT vs. BE.HGD + EAC *TP53* MUT. Isoform significantly linked with cancer-specific mortality in **C** BE.LGD vs. BE.HGD + EAC and **D** BE.LGD *TP53* WT vs. BE.HGD + EAC *TP53* MUT. *P*-values were determined using the Cox proportional hazards model. Each bar represents a 95% confidence interval.

Next, survival analysis was performed to identify IS significantly linked with patient survival based on individual isoform fraction (IF) determination. As shown in Fig. 2A, 42 isoforms are significantly linked with all-cause patient mortality comparing BE.LGD to BE.HGD + EAC. Isoforms with a positive hazard ratio represent isoforms with elevated IF in BE.HGD + EAC vs. BE.LGD groups that are associated with reduced patient survival, whereas isoforms with a negative hazard ratio are isoforms that when reduced, are correlated with worse patient survival. As shown in Fig. 2A, higher IF of *SMIM6* isoform (ENST00000556126), *C1orf54* isoform (ENST00000369098), *CFDP1* isoform (ENST00000283882), *TPM4* isoform (ENST00000653979), and *TTLL12* isoform (ENST00000216129) are among the top isoforms with increased IF significantly correlated with poor patient survival. A similar number of isoforms (*n* = 39 isoforms) are significantly linked with patient survival when *TP53* mutation status is included in the analysis (Fig. 2B). Isoforms including *HM13* isoform (ENST00000340852), *CFDP1* isoform (ENST00000283882), *DANT2* isoform (ENST00000430756), and *C1orf54* isoform (ENST00000369098) show higher IF in BE.HGD + EAC *TP53* MUT group, which were also significantly linked with worse patient survival. Next, survival analysis was performed to identify IS significantly linked with cancer-specific patient mortality (Fig. 2C, D). Forty and twenty-six isoforms were significantly linked with patient survival in BE.LGD vs. BE.HGD + EAC and BE.LGD *TP53* WT vs. BE.HGD + EAC *TP53* MUT, respectively. Although the number of isoforms significantly linked with patient survival was similar between all-cause and cancer-specific mortalities in BE.LGD vs. BE.LGD + EAC, the number of isoforms significantly linked with cancer-specific mortality (*n* = 26 isoforms) was lower than the number of isoforms linked with all-cause mortality in BE.LGD *TP53* WT vs. BE.HGD + EAC *TP53* MUT (*n* = 39 isoforms). Venn diagrams further reveal survival-linked isoforms that are common and unique to each comparison group (Fig. S1).

To gain mechanistic insight and investigate whether isoforms can be therapeutically targeted, two isoforms, *TTLL12* (ENST00000216129) and *HM13* (ENST00000340852), were chosen for the genetic knockdown studies in EAC cell lines. Both isoforms are the coding isoform of the gene and show significantly higher IF in BE.HGD + EAC group and significantly linked with reduced patient survival. Specifically, the *TTLL12* isoform was among the top isoforms identified to be associated with all-cause patient mortality in BE.LGD vs BE.HGD + EAC group, whereas the *HM13* isoform was among the top isoforms identified in BE.LGD *TP53* WT vs. BE.HGD + EAC *TP53* MUT (both all-cause and cancer-specific mortality). In addition, IS events of both genes were validated using qRT-PCR, as shown in Fig. 1D.

To further validate the potential oncogenic role of *TTLL12-201* and *HM13-201* in EAC, we performed RNA-seq analysis of esophageal samples collected from rats with reflux-driven EAC, as we previously published [12]. A significant upregulation of *Ttll12-201* was observed in rats with reflux-driven EAC (Fig. S2A), compared to water controls. Similarly significant upregulation of the *HM13* ortholog, *Mcts2*, was also observed, although the exact ortholog of *HM13-201* in rats is unknown (Fig. S2B). We also performed RNA-seq analysis of EAC samples from a human cohort in the Cancer Genome Atlas Program (TCGA) [13], revealing significant upregulation in *TTLL12-201* (Fig. S2C) and *HM13-201* in EAC samples, compared to normal tissues (Fig. S2D).

### Isoform-specific knockdown of *TTLL12* inhibited EAC cell viability and migration via chaperone-mediated autophagy

**Fig. 3:**
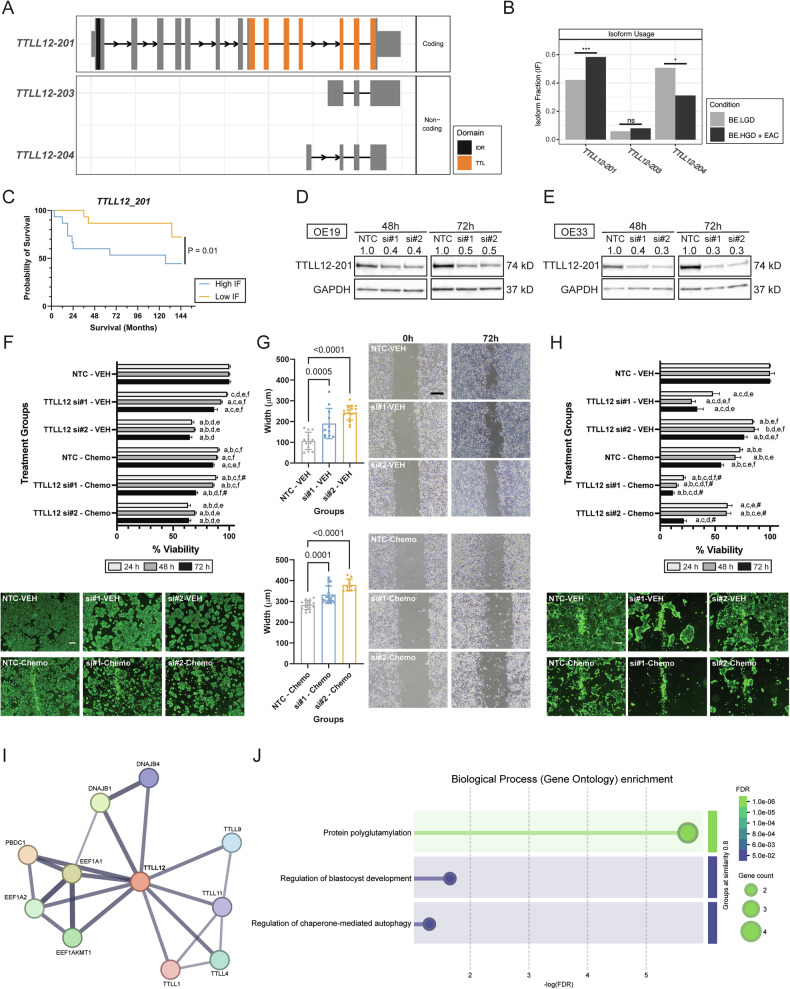
Knockdown of the *TTLL12-201* isoform inhibited OE19 and OE33 EAC cell viability and migration. **A** Isoform switching of *TTLL12*. **B** Isoform fractions of *TTLL12* comparing BE.LGD to BE.LGD + EAC. **C** Kaplan–Meier survival curve indicating significant survival differences based on the *TTLL12-201* isoform fraction. **D** Immunoblot showing knockdown of *TTLL12-201* in OE19 EAC cells using siRNA. **E** Immunoblot showing knockdown of *TTLL12-201* in OE33 EAC cells using siRNA. **F** Viability results and representative fluorescent images (72 h) of OE19 cells treated with siRNA alone or in combination with chemotherapy agents (*n* = 4–6/group). **G** Migration assay results and representative microscopic images of OE19 cells treated with siRNA alone or in combination with chemotherapy agents (*n* = 10–20/group). **H** Viability results and representative fluorescent images (72 h) of OE33 cells treated with siRNA alone or in combination with chemotherapy agents (*n* = 4–6/group). **I** Protein interaction prediction network of TTLL12 determined by STRING analysis. **J** Biological processes result from enrichment analysis of TTLL12. Error bars represent the standard error of the mean. Significance was determined using ANOVA with Tukey’s post-hoc test for multiple comparisons between treatments. Within each time point, treatments were significantly different from a = NTC-VEH, b = siRNA#1–VEH, c = siRNA#2–VEH, d = NTC–Chemo, e = siRNA#1–Chemo, f = siRNA#2–Chemo. Pound sign (#) denotes synergy. Synergy was calculated using the Combination Index equation. IF isoform fraction, NTC non-targeting control, Scale bar, 200 μm; Chemo, combination of paclitaxel and carboplatin.

IS of *TTLL12* was observed between two of its isoforms, *TTLL12-201* (ENST00000216129) and *TTLL12-204* (ENST00000494035) (Fig. 3A). *TTLL12-201* is the coding isoform of *TTLL12*, containing several tubulin tyrosine ligase (TTL) domains, whereas *TTLL12-204* is a predicted non-coding isoform (Fig. 3A). *TTLL12-201* has a significantly higher IF in BE.HGD + EAC group and *TTLL12-204* showed a decreased IF in this group, supporting a significant IS (Fig. 3B). As shown in Figs. 3C and S3B, a higher IF of *TTLL12-201* significantly decreases overall patient survival (44.4% survival probability versus 72.2%), linking *TTLL12-201* to overall survival among EAC patients for the first time. In addition, gene-level expression of *TTLL12* is significantly lower in BE.HGD + EAC compared to BE.LGD (Fig. S3A).

To investigate the therapeutic potential of targeting *TTLL12-201*, two EAC cell lines, OE19 and OE33, were treated with two isoform-specific siRNAs targeting *TTLL12-201*. As shown in Figs. 3D, E, and S4, isoform-specific knockdown of *TTLL12-201* led to a 50–70% reduction in TTLL12-201 isoform and protein expression levels across cell lines over time, supporting successful knockdown using both siRNAs. Results of the viability assay in OE19 cells (Fig. 3F) reveal that treatment with either of the *TTLL12-201* siRNAs led to significant inhibition of OE19 cell viability at 48 h (69–93%) and 72 h (65–87%), respectively. Additionally, synergy, reported as the combination index, was observed for OE19 cells treated with both siRNA#1 and chemotherapy agents at 24 h and 72 h. A migration assay performed in OE19 cells (Fig. 3G) reveals that siRNA knockdown alone (siRNA#1: 190 ± 73.3 μm and siRNA#2: 242 ± 35.4 μm) or in combination with chemotherapy agents (siRNA#1+Chemo: 333 ± 41.8 μm and siRNA#2+Chemo: 379 ± 28.5 μm) significantly inhibits cell migration compared to cells treated with non-targeting control (NTC) (107 ± 41.4 μm) or chemotherapy agents alone (283 ± 21.9 μm) at 72 h. Similar but more substantial effects were also observed in OE33 cells. As shown in Fig. 3H, *TTLL12-201* isoform knockdown led to significant inhibition of OE33 cell viability at all three time points. At 72 h, siRNA treatment alone reduced OE33 cell viability to 34–74%. Synergistic effects were observed at all three time points for combination treatments of individual siRNAs and chemotherapy agents (Fig. 3H). At 72 h, the combination treatment decreased OE33 cell viability to 12–22%. *TTLL12-201* knockdown also led to significant inhibition of OE33 cell migration (Fig. S5). The results indicate that *TTLL12-201* knockdown sensitized EAC cells to chemotherapeutic agent-induced cell death.

### Isoform-specific *TTLL12* knockdown induces chaperone-mediated autophagy (CMA)

**Fig. 4:**
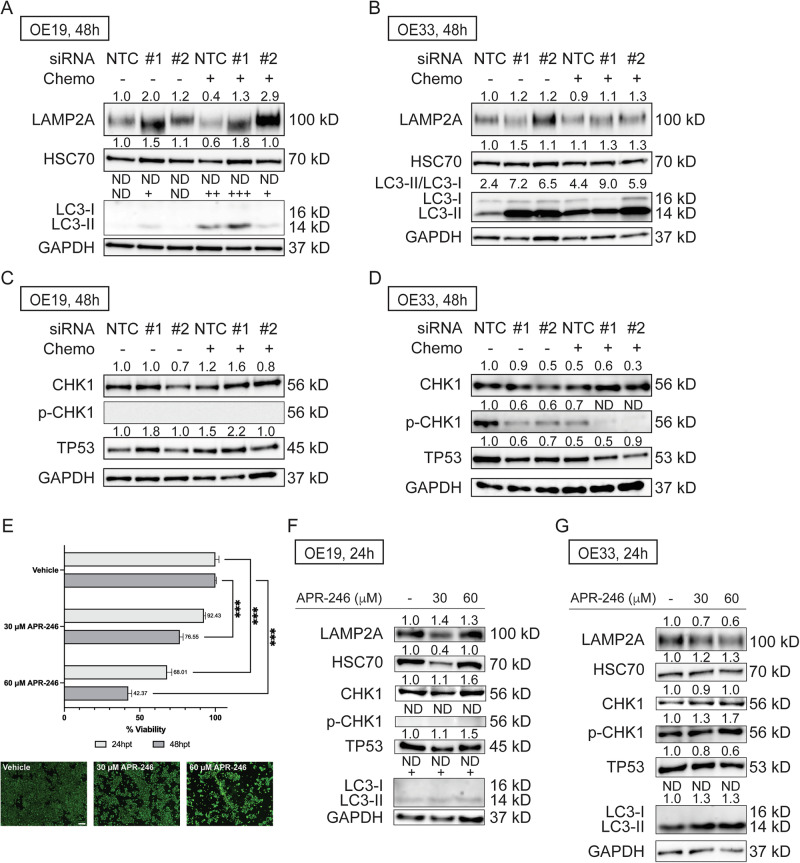
*TTLL12-201* knockdown induces chaperone-mediated autophagy (CMA) in OE19 and OE33 cells. Immunoblot of CMA markers, LAMP2A, and HSC70, in **A** OE19 and **B** OE33 EAC cell lines. Immunoblot of CHK1 and TP53 in **C** OE19 and **D** OE33 cells following *TTLL12-201* siRNA knockdown alone or in combination with chemotherapy agent treatment. **E** Viability results and representative fluorescent images (48 h) of OE19 cells treated with APR-246. Immunoblot of CMA markers, CHK1, and TP53 in **F** OE19 and **G** OE33 cells following APR-246 treatment. NTC non-targeting control, Scale bar, 200 μm; ***, *P* < 0.001.

To investigate potential mechanisms linked with *TTLL12* isoform knockdown, a STRING protein interaction prediction was performed (Fig. 3I, J). Biological process analysis of the TTLL12 protein interaction network reveals significant biological processes linked with protein polyglutamylation, regulation of blastocyst development, and regulation of CMA (Fig. 3J). Considering altered CMA is reported in esophageal squamous cell carcinoma (ESCC) [14], immunoblotting of two chaperone-mediated autophagy markers, LAMP2A and HSC70, was performed. Results show upregulation of LAMP2A and HSC70 upon *TTLL12-201* knockdown in both OE19 and OE33 cells (Fig. 4A, B), with more substantial induction in OE19 cells. *TTLL12* isoform knockdown also resulted in potent induction of autophagy-linked LC3-II in OE33 cells and minor LC3-II induction in OE19 cells, where LC3-I has low baseline expression levels, consistent with previous reports of differential capacity for autophagy induction in EAC cells [15]. Immunoblotting of CHK1, phospho-CHK1 (Ser345), and TP53 was also performed (Fig. 4C, D) because CMA targets both CHK1 and missense mutant TP53 for degradation [16, 17]. Results showed decreased expression of CHK1 in both OE19 and OE33 cells. Moreover, decreased expression of phospho-CHK1 and TP53 was observed in OE33 cells but less evident in OE19 cells, as they carry a frameshift mutation resulting in expression of only the truncated form of TP53 (45 kD).

To further validate that targeting mutant TP53 leads to EAC cell death, OE19 cells were treated with APR-246, a pharmacological agent that targets mutant TP53 and induces autophagy in cancer cells [18]. As shown in Fig. 4E, treatment of APR-246 led to a significant decrease in cell viability at 24 h and 48 h in OE19 cells, and, as we previously reported, a significant decrease in cell viability in OE33 cells following APR-246 treatment [11]. Immunoblots of LAMP2A and HSC70 suggest that APR-246 treatment increased LAMP2A expression in OE19 cells and HSC70 expression in OE33 cells, suggesting activation of CMA (Fig. 4F, G). Similar to *TTLL12-201* siRNA-induced protein modulation, decreased expression of TP53 and increased expression of autophagic LC3-II were observed in OE33 cells (Fig. 4G). Although modulation of CHK1 and/or phospho-CHK1 was not observed in either EAC cell line at 24 h post-treatment, inhibition was observed at 48 h post-treatment, with more substantial changes noted in OE33 cells (Fig. S6).

### Isoform-specific knockdown of *HM13* inhibited EAC cell viability and migration through the unfolded protein response pathway

**Fig. 5:**
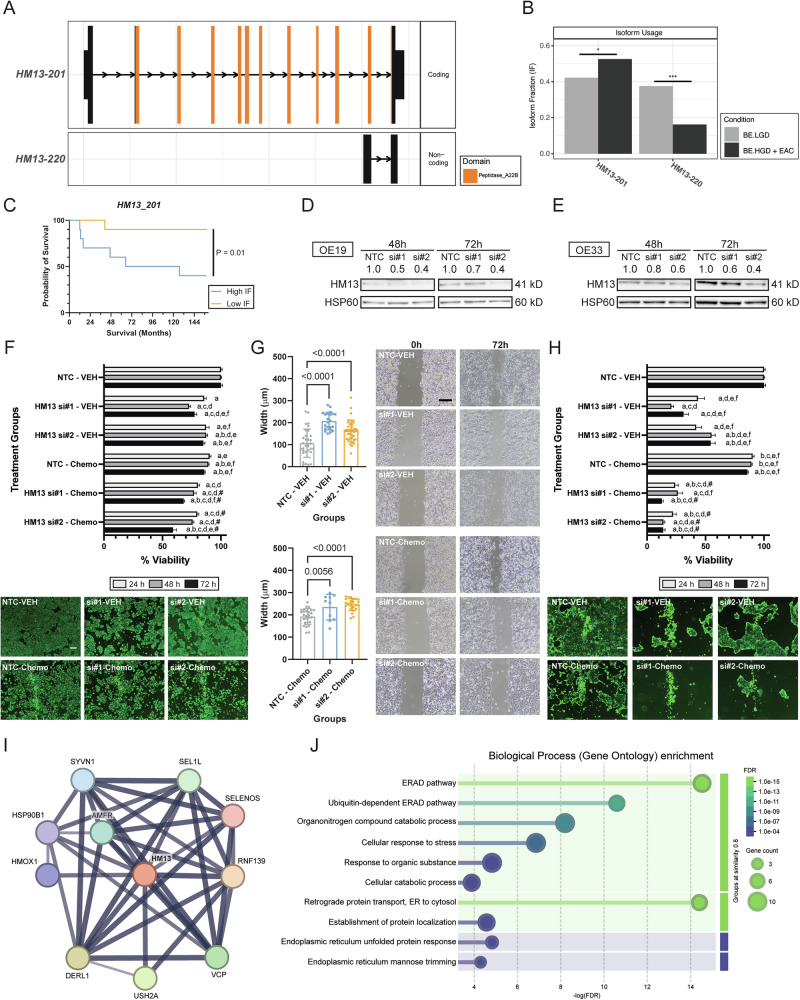
Knockdown of the *HM13-201* isoform inhibited OE19 and OE33 EAC cell viability and migration. **A** Isoform switching of *HM13*. **B** Isoform fractions of *HM13* comparing BE.LGD to BE.LGD + EAC. **C** Kaplan-Meier survival curve indicating significant survival differences based on the *HM13-201* isoform fraction. **D** Immunoblot showing knockdown of *HM13-201* in OE19 cells using siRNA. **E** Immunoblot showing knockdown of *HM13-201* in OE33 cells using siRNA. **F** Viability results and representative fluorescent images (72 h) of OE19 cells treated with siRNA alone or in combination with chemotherapy agents (*n* = 4-6/group). **G** Migration assay results and representative microscopic images of OE19 cells treated with siRNA alone or in combination with chemotherapy agents (*n* = 10–36/group). **H** Viability results and representative fluorescent images (72 h) of OE33 cells treated with siRNA alone or in combination with chemotherapy agents (*n* = 4-6/group). **I** Protein interaction prediction network of HM13 determined by STRING analysis. **J** Biological processes result from enrichment analysis of HM13. Error bars represent the standard error of the mean. Significance was determined using ANOVA with Tukey’s post-hoc test for multiple comparisons between treatments. Within each time point, treatments were significantly different from a = NTC-VEH, b = siRNA#1–VEH, c = siRNA#2–VEH, d = NTC–Chemo, e = siRNA#1–Chemo, f = siRNA#2–Chemo. Pound sign (#) denotes synergy. Synergy was calculated using the combination index equation. IF isoform fraction, NTC non-targeting control, Scale bar, 200 μm; Chemo, combination of paclitaxel and carboplatin.

*HM13* IS was observed between BE.HGD + EAC and BE.LGD. Specifically, IS of *HM13* was observed between *HM13-201* (ENST00000340852) and *HM13-220* (ENST00000493364) (Fig. 5A). *HM13-201* is one of the coding isoforms of *HM13*, containing several signal peptide peptidase domains, whereas *HM13-220* is one of the non-coding isoforms of *HM13* (Fig. 5A). Similar to *TTLL12-201*, the *HM13-201* isoform is significantly elevated in BE.HGD + EAC patient samples compared to *HM13-220*, which shows a significantly reduced IF (Fig. 5B). Higher levels of *HM13-201* IF are significantly linked with reduced patient survival (40.0% survival probability vs. 90.0%, Figs. 2B, 5C, and S7B), supporting its role in EAC. Overall, gene-level expression of *HM13* is also significantly lower in BE.HGD + EAC compared to BE.LGD (Fig. S7A).

OE19 and OE33 cells were similarly treated with isoform-specific siRNAs targeting *HM13-201*, and immunoblots confirmed the successful knockdown in both cell lines at 48 h and 72 h (Fig. 5D, E). As shown in Fig. 5F, *HM13-201* knockdown significantly decreases OE19 cell viability at 24 h, 48 h, and 72 h. At 72 h, siRNA treatment alone reduced OE19 cell viability to 77–85%. Moreover, synergistic effects were observed for siRNA#1 at 48 h and 72 h and for siRNA#2 at 24–72 h. At 72 h, the combination treatment of siRNA#1 and chemo drugs reduced OE19 cell viability to 68%, and the combination treatment of siRNA#2 and chemo drugs decreased cell viability to 59%, supporting that *HM13* isoform knockdown sensitizes OE19 cells to chemotherapy agents. Results of migration assays evaluating OE19 cells upon siRNA treatment, shown in Fig. 5G, reveal that *HM13* isoform knockdown significantly inhibits OE19 cell migration compared to cells treated with NTC or chemotherapy agents alone. Similar results in cell viability were also observed in OE33 cells (Fig. 5H). *HM13-201* knockdown significantly inhibited OE33 cell viability, with synergistic effects observed with both siRNA#1 and siRNA#2 treatments combined with chemotherapy agents. At 72 h, siRNA#1 combined with chemo drugs decreased OE33 cell viability to 13%, and siRNA#2 combined with chemo drugs decreased cell viability to 14%. Similar to results in OE19 cells, *HM13-201* knockdown also significantly inhibited the migration capability of OE33 cells (Fig. S8).

### Isoform-specific knockdown of *HM13* activates unfolded protein response (UPR) and halts protein translation

**Fig. 6:**
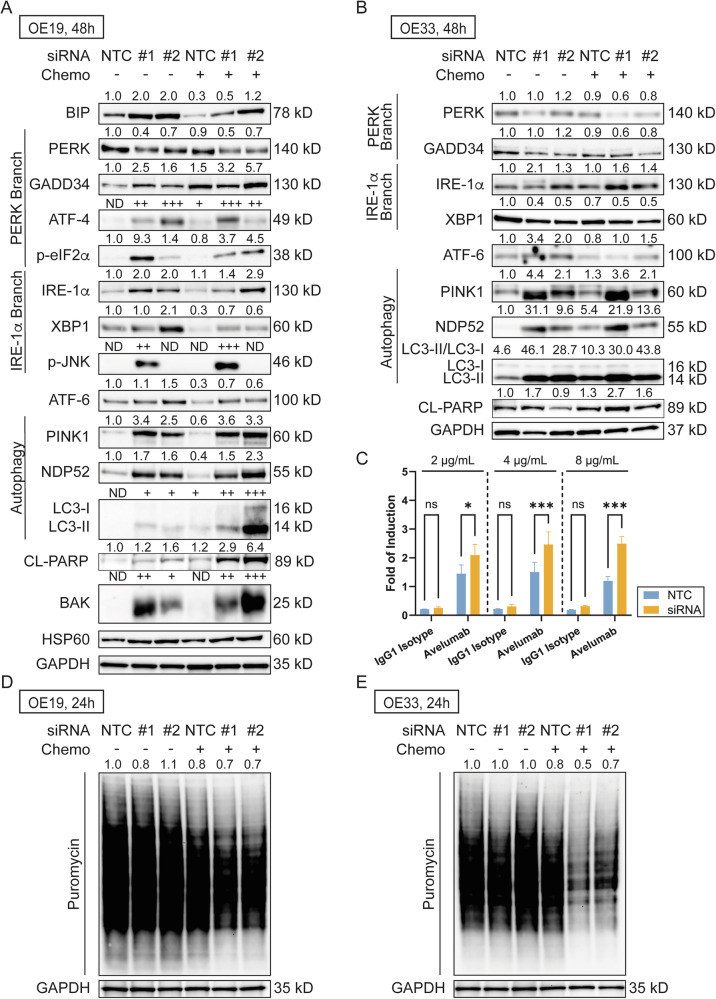
*HM13-201* knockdown induces unfolded protein response (UPR) in EAC cell lines and increases response to avelumab, an anti-PD-L1 agent. Immunoblot of key UPR proteins in **A** OE19 and **B** OE33 cells. **C** *HM13-201* knockdown increases response to avelumab in OE33 cells (*n* = 3/group). Error bars represent the standard error of the mean. SUnSET assay of **D** OE19 and **E** OE33 cells showing inhibition of protein translation. NTC non-targeting control, ND not detected, CL cleaved protein, ns non-significant, *, *P* < 0.05; ***, *P* < 0.001.

A STRING protein interaction prediction reveals interacting proteins of HM13 and related biological processes (Fig. 5I, J), identifying multiple pathways associated with the endoplasmic reticulum (ER)-associated protein degradation (ERAD) pathway and ER UPR pathway. The UPR consists of three main branches: PERK, IRE-1α, and ATF-6 [19]. Immunoblotting of key UPR-related proteins was performed in both OE19 (Fig. 6A) and OE33 cells (Fig. 6B). In OE19 cells, increased expression of BIP, phosphorylation of PERK (as indicated by an upshift of the PERK band), and activation of downstream target proteins (GADD34, ATF-4, and phospho-eIF2α) were observed upon *HM13-201* knockdown alone and in combination with chemotherapy agents, compared to NTC or chemotherapy treatment alone. Similar induction of IRE-1α and its downstream targets XBP1 and phospho-JNK, and ATF-6 was also observed. The UPR is also documented to induce autophagy and apoptosis [19]. Expression of autophagy markers (PINK1, NDP52, and LC3-II) and the apoptosis marker BAK and cleaved PARP were increased upon *HM13-201* knockdown alone and in combination with chemotherapy agents, compared to NTC, with modest reductions with chemotherapy treatment alone. In contrast, in OE33 cells, the PERK branch was not activated. Expression of IRE-1α was increased upon *HM13* knockdown, but not its downstream factor XBP1, and ATF-6 was increased upon *HM13* knockdown. *HM13* knockdown in OE33 cells also resulted in increased expression of the autophagy-related markers, PINK1, NDP52, and LC3-II, and the apoptosis marker, cleaved PARP. Interestingly, OE33 cells did not express BIP, phospho-eIF2A, or phospho-JNK in contrast to OE19 cells (data not shown). In addition, because studies of HM13 in other cancers suggest an association with an immunosuppressive microenvironment and PD-1 expression [20, 21], an antibody-dependent cell-mediated cytotoxicity (ADCC) assay was performed to evaluate whether *HM13-201* knockdown increases response to the anti-PD-L1 agent, avelumab, in EAC cells. A significantly increased response to avelumab is observed in OE33 cells upon *HM13* knockdown, compared to cells treated with NTC followed by avelumab (Fig. 6C). However, such a response was not observed in OE19 cells. Since the UPR also leads to translation arrest [19], SUnSET assays were performed to measure changes in protein translation. Both OE19 (Fig. 6D) and OE33 (Fig. 6E) cells show decreased protein translation upon *HM13-201* knockdown. A stronger magnitude of effect was evident in cells treated with *HM13* siRNA combined with chemotherapy agents.

**Fig. 7:**
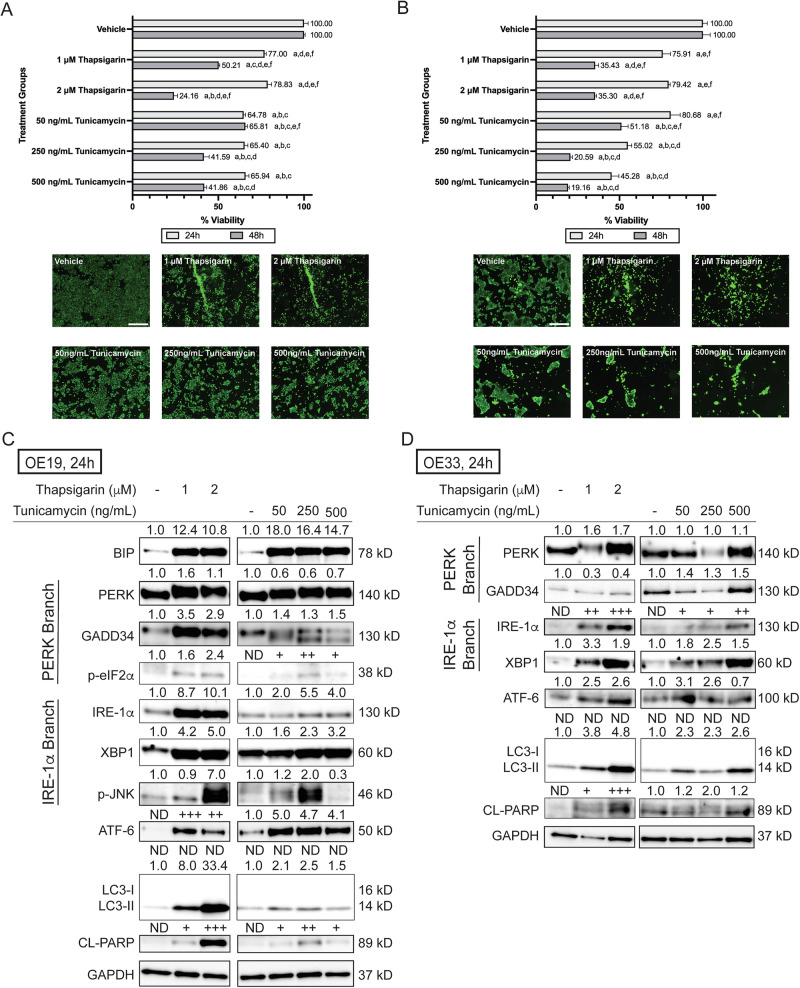
Treatment of pharmacological unfolded protein response (UPR) inducers, thapsigarin and tunicamycin, leads to OE19 and OE33 cell death. Viability results and representative fluorescent images (48 h) of **A** OE19 and **B** OE33 cells treated with thapsigarin and tunicamycin. Error bars represent the standard error of the mean. Immunoblot of key UPR proteins and their downstream factors in **C** OE19 and **D** OE33 cells, following thapsigarin or tunicamycin treatment. Significance was determined using ANOVA with Tukey’s post-hoc test for multiple comparisons between treatments. Within each time point, treatments were significantly different from a = Vehicle, b = 1 μM thapsigarin, c = 2 μM thapsigarin, d = 50 ng/mL tunicamycin, e = 250 ng/mL tunicamycin, f = 500 ng/mL tunicamycin. CL cleaved protein. Scale bar, 500 μm.

To further validate the causality that *HM13-201* knockdown led to activation of UPR followed by EAC cell death induction, OE19 and OE33 cells were treated with pharmacological agents, thapsigarin and tunicamycin, that induce UPR [22, 23]. As shown in Fig. 7A, both thapsigarin and tunicamycin treatment significantly decreases the viability of OE19 cells at 24 h and 48 h. At 48 h, 2 μM thapsigarin treatment decreased OE19 cell viability to 24.16%, and 500 ng/mL tunicamycin treatment decreased OE19 cell viability to 41.86%. Similarly, thapsigarin and tunicamycin potently inhibited OE33 cell viability in a time and dose-dependent manner (Fig. 7B). At 48 h, 2 μM thapsigarin treatment decreased OE33 cell viability to 35.30%, and 500 ng/mL tunicamycin treatment decreased OE33 cell viability to 19.16%. Immunoblotting was also performed on key UPR proteins and their downstream factors. Pharmacological-agent-induced UPR shows a similar trend compared to UPR induced by *HM13-201* siRNA treatment in both OE19 and OE33 cells. In OE19 cells (Fig. 7C), thapsigarin or tunicamycin treatment increases the expression of BIP, phosphorylation of PERK (based upon altered migration), and expression of downstream factors of the PERK branch (GADD34 and phospho-eIF1α). In addition, increased expression of IRE-1α and its downstream factors (XBP1 and phospho-JNK) was observed, as well as increased expression of activated or cleaved ATF-6 (50kD). Lastly, thapsigarin or tunicamycin treatment also led to increased expression of LC3-II and cleaved PARP, suggesting activation of programmed cell death and autophagy following agent treatment. In OE33 cells (Fig. 7D), thapsigarin treatment led to phosphorylation of PERK, as did high concentrations of tunicamycin. Similar to HM13-201 siRNA-induced UPR, increased expression of IRE-1α and its downstream factor XBP1 was observed, as well as induction of ATF-6, LC3-II, and cleaved PARP in OE33 cells following thapsigarin or tunicamycin treatment. Taken together, the results suggest that UPR, induced by either *HM13-201* knockdown or pharmacological agents, can lead to cell death in EAC cells via apoptosis and autophagy.

### Isoform-specific knockdown of *TTLL12* and *HM13* had minimal effect on normal esophageal epithelial cells

To investigate whether isoform-specific knockdown of *TTLL12* and *HM13* has cytotoxic effects in normal esophageal epithelial cells, Het-1A cells were treated with isoform-specific *TTLL12* and *HM13* siRNAs. Results from cell viability assays indicated that knockdown of *TTLL12-201* led to a 2–4% decrease in Het-1A cell viability and knockdown of the *HM13-201* led to a 3–7% decrease in Het-1A cell viability (Fig. S9), suggesting minimal cytotoxicity associated with targeting these isoforms in normal cells.

## Discussion

This research sought to investigate IS events linked to EAC patient survival, reveal mechanisms associated with dysregulation of specific isoforms, and study whether isoforms may be targeted for improved therapeutic response in EAC. Specifically, IS analysis was performed using RNA-seq data from EAC and Barrett’s Esophagus (BE) precursor lesions to identify IS events during pathological progression that are significantly linked with patient survival, and in vitro studies were conducted to explore mechanisms implicated in IS in the context of EAC. We identified 42 and 40 IS significantly linked to all-cause and cancer-specific mortality, respectively. A number of isoforms involved in metabolic reprogramming and drug resistance were uniquely linked to cancer-specific mortality, including *KRAS* and *SLC28A3* [24, 25]. Given the role of *TP53* mutation in EAC progression, we further stratified results based on *TP53* mutation status, identifying 39 and 26 isoforms associated with all-cause and cancer-specific mortality, respectively (Fig. 2).

Among the survival-linked isoforms we identified in this cohort, IS of *TPM4*, *TTLL12*, *COL4A2*, *IGSF3*, *FSIP1*, *RPL22L1*, and *KRAS* have been previously reported in other cancer tissues or cell lines [26–33]. In EAC, *RNF128* is the only isoform-switched gene that has been broadly studied [34]. Herein, we selected two isoforms identified based on survival analysis, *TTLL12-201* and *HM13-201*, and performed isoform-specific siRNA knockdown followed by a series of in vitro studies. The results showed that knockdown of *TTLL12* and *HM13* isoforms significantly inhibited the viability of two EAC cell lines, sensitized EAC cell lines to standard-of-care chemotherapy agents, and inhibited cell migratory potential. Importantly, the knockdown of both induced isoforms had minimal effects on a normal esophageal cell line (Het-1A), suggesting both isoforms may be therapeutically targeted in EAC with minimal side effects.

TTLL12, also known as tubulin tyrosine ligase 12, is one of the most understudied proteins in the TTL/TTLL family. Overexpression of gene-level *TTLL12* is documented in various cancers, including gastric adenocarcinoma, lung adenocarcinoma, colorectal cancer (CRC), and prostate cancer [27, 35–38]. IS of *TTLL12* is also observed in ESCC cell lines compared to normal esophageal Het-1A cells [27]. In CRC, an alternate promoter leads to IS of *TTLL12* compared to normal tissues. In our study, we observe significantly higher isoform usage of a coding transcript of *TTLL12* (*TTLL12-201*) in HGD and EAC compared to LGD tissues (Fig. 3). Isoform-specific knockdown of this isoform significantly decreased the viability and cell migration potential of two EAC cell lines. Additionally, isoform-specific knockdown sensitized both cells to standard-of-care chemotherapy agents. STRING analysis and immunoblot support that TTLL12-201 is linked with CMA. TTLL12 is speculated to be a negative regulator in inflammation-driven nitrosative stress, which inhibits nitrotyrosine-induced cytotoxicity in malignant cells. In non-cancerous cells, nitrotyrosine can induce cell death through multiple mechanisms, including apoptosis and autophagy. However, in cancer cells, TTLL12 decreases the cytotoxicity of nitrotyrosine, potentially by inhibiting nitrotyrosination of α-tubulin [39–41]. EAC is also an inflammation-driven cancer, and nitrosative stress is reportedly linked with EAC progression [42–44], raising the possibility that upregulation of the coding transcript of *TTLL12* in HGD and EAC suppresses cell death. The role of CMA in EAC remains underexplored. In other cancers, CMA exerts both anti-tumor and pro-tumor activities [45]. The anti-tumor effect of CMA is largely achieved by targeted degradation of cancer promoters, genome quality control, and influencing glucose metabolism [45]. For example, activated CMA is known to target missense mutant TP53 for degradation and inhibit CHK1 to prevent DNA damage response [16, 17]. In EAC, over 80% of patients carry a *TP53* mutation, and a CHK1/2 inhibitor, AZD7762, potently inhibits viability of an EAC cell line [8, 10]. EAC cell lines used in our study, OE19 and OE33, carry a frameshift and a missense *TP53* mutation, respectively [46, 47]. The reason why activation of CMA fails to degrade TP53 in OE19 is likely because OE19 carries a frameshift *TP53* mutation. We also treated both EAC cells with a pharmacological agent that targets mutant TP53 and induces autophagy, which revealed similar trends in loss of cell viability and protein modulation as observed in EAC cells treated with *TTLL12-201* siRNA. Our results align with published studies reporting that activation of CMA leads to CHK1 inhibition and degradation of missense mutant TP53, further supporting the therapeutic potential of targeting *TTLL12-201* in EAC.

HM13, also known as histocompatibility minor 13, is linked with poor prognosis and patient survival, metastasis, and cancer proliferation and migration in hepatocellular carcinoma, breast cancer, and CRC [20, 21, 48–50]. As an ER-resident protein, HM13 plays a role in the UPR [51]. ER stress induces activation of the UPR, which either leads to recovery of ER function or ER-stress-induced cell death if cells fail to overcome ER stress [19]. In cancer, two distinct routes have been proposed to target the UPR: to trigger UPR-mediated cell death or block ER recovery mechanisms [52]. In EAC, whether the UPR could be therapeutically targeted remains poorly understood. One study shows that EAC cell lines have higher expression levels of BIP, PERK, IRE-1α, and XBP1, as compared to normal esophageal cells, suggesting a vulnerability [53]. However, the latter study did not measure expression levels of downstream factors of UPR. Herein, we measured expression levels of downstream factors of UPR, including GADD34, phospho-JNK, PINK1, NDP52, LC3, PARP, and BAK, to determine whether isoform-specific knockdown of *HM13-201* can trigger UPR-mediated cell death in EAC cell lines. In OE19 cells, elevated expression of phospho-JNK and BAK (UPR-mediated apoptosis markers [19]), and cleaved PARP was observed, as well as increased expression of LC3-II, PINK1, and NDP52, implying activation of mitophagy in OE19 cells. Mitophagy can trigger cell death, both dependent (via BAK) and independent of apoptosis [54]. However, additional studies are required to delineate the specific crosstalk between UPR and mitophagy. In contrast, multiple UPR makers were not expressed in OE33 cells, likely due to the molecular heterogeneity of EAC. However, activation of mitophagy and inhibition of global translation were observed in both EAC cell lines, suggesting that alternative UPR pathways likely exist in OE33 cells. We also treated both EAC cells with pharmacological agents, thapsigarin and tunicamycin, that induce UPR. The results further support our observation that activation of UPR can lead to EAC cell death. Moreover, one study in hepatocellular carcinoma showed that HM13 is a predictive biomarker for immunotherapy [20]. The ADCC assay result in our study suggests that isoform-specific *HM13* knockdown increased the response to avelumab, an anti-PD-L1 agent, in OE33 cells, pointing to a potential linkage between *HM13-201* and response to immunotherapy in EAC. This finding is of potential importance considering EAC has a low response rate to PD-1/PD-L1 immune checkpoint inhibitors [55]. OE19 has a significantly lower PD-L1 expression compared to OE33 and did not respond to avelumab treatment [56].

This research provides new insight regarding genes that are isoform switched with pathological progression to EAC and are associated with patient mortality, suggesting that specific isoforms may be targeted for enhanced therapeutic or prognostic value. Overall, genetic knockdown of two of the top mortality-linked isoforms (*HM13* and *TTLL12* isoforms) revealed that isoforms may be therapeutically targeted to sensitize EAC to chemotherapy agents or immunotherapy with minimal cytotoxicity in normal esophageal cells. Generalizability of the current study is unknown because, despite our comprehensive efforts to search the public and permission-based databases for external validation datasets, we could not identify any parallel datasets containing sufficient BE patient samples with stratification by grade of dysplasia and *TP53* mutation status among EAC progressors. Additionally, most of the datasets we identified lacked the depth of sequencing required to perform isoform switching analysis and were further hindered by small sample sizes in subgroups (i.e., BE with dysplasia). Future studies will evaluate the potential of using small molecules or splice-switching antisense oligonucleotides to target isoforms in EAC [57, 58]. In addition, we plan to further identify specific IS driver events associated with EAC progression using multi-omics approaches, such as long-read RNA-seq, proteomics, and functional validation. Research in a larger cohort of EAC progressors with multiple biopsies acquired from patients over time, or comparing EAC progressors to non-progressors would be informative.

## Material and methods

### Isoform switching analysis of patient RNA-seq data

IS analysis was performed on patient RNA-seq data derived from esophageal tissues, as we previously reported [11]. Kallisto (version 0.46.2) was used to quantify transcripts in the transcriptomics data [59]. IsoformSwitchAnalyzeR (version 1.14.1) was applied in R (version 4.1.1) to characterize IS events [60, 61]. Briefly, IS events were determined by calculating isoform fractions (IFs) and differential isoform fractions (dIFs) between BE.LGD esophageal samples (*n* = 17) and BE.HGD + EAC esophageal samples (*n* = 28) alone or in combination with *TP53* mutation status. IFs were calculated by dividing specific isoform expression by total gene expression for a given pathology, and dIFs were determined by calculating the difference of IFs between BE.LGD and BE.HGD + EAC alone or in combination with *TP53* mutation status. Significant isoform switching events were determined using an absolute dIF cutoff of 0.1 and an FDR cutoff at 0.05, as determined by the Mann–Whitney *U*-test [60]. In addition, several external analyses were performed to identify significant isoform switching events with predicted functional consequences, including prediction of coding potential using CPAT, signal peptides using SignalP, intrinsically disorder regions using IUPred2A, coding domains using Pfam, and sensitivity to nonsense-mediated decay [62–65]. The Venn diagrams in Figs. 1A and S1 were generated using InteractiVenn, and dot plots summarizing isoform switching analysis results in Fig. 1B, C were generated using ggplot2 in R [66, 67]. Individual isoform switching plots (Figs. 3A, B and 5A, B) were generated from the IsoformSwitchAnalyzeR package. Protein interaction prediction (Figs. 3I and 5I) and Biological Process enrichment analysis (Figs. 3J and 5J) were performed using the STRING database [68].

### Quantification of isoform expression using qRT-PCR

Quantification of isoform expression using qRT-PCR was performed using RNA extracted from the same patient cohort, as previously described [11]. Isoform-specific primers of *ATP9B*, *HM13*, *KIF16B*, *TRIM29*, and *TTLL12* were designed using the NCBI Primer-BLAST [69]. Isoform-specific primer sequences of *TRIM29* were previously published, and primer sequences of *ATP9B*, *HM13*, *KIF16B*, and *TTLL12* isoforms or genes are reported in Table S1 [11]. Primers for qRT-PCR were synthesized by Eurofins Scientific (Louisville, KY, USA). qRT-PCR was performed using SsoAdvanced Universal SYBR Green Supermix with CFX Connect Thermo Cycler (Bio-Rad Laboratories, Hercules, CA, USA). Relative isoform expression was determined using *GAPDH* as the housekeeping gene, and IF was calculated similarly to the IsoformSwitchAnalyzeR package.

### Patient survival analysis

To identify isoforms significantly linked with patient survival, IF of isoform-switched genes was calculated for each patient using TPM values, and patients were stratified based on IF into tertiles. Survival analysis was performed by comparing patients sorted into the high tertile isoform usage group to the low tertile isoform usage group using the Cox proportional hazards model (survival package, version 3.5-5) in R [70]. Cancer-specific mortality was determined by only including patients whose cause of death was related to esophageal cancer, with all other patients censored or not counted as endpoints, but removed from the at-risk group in the same manner that patients who are lost to follow-up are removed [71]. Selection bias was minimized by using an unbiased, genome-wide approach to identify IS events using isoformSwitchAnalyzeR, and survival analysis was performed for all isoforms that are significantly switched.

### Cell culture

OE19 and OE33 EAC cell lines were cultured in RPMI 1640 medium with 2.0 mM L-glutamine, 10^4^ units/mL penicillin, 10^4^ μg/mL streptomycin, 1 mM sodium pyruvate, and 10% fetal bovine serum (FBS), as previously reported [72]. Cell culture reagents were acquired from ThermoFisher Scientific (Waltham, MA, USA). The cell lines were maintained as monolayers and incubated at 37 °C with 5% CO_2_. Both cell lines were acquired from the European Collection of Authenticated Cell Cultures (ECACC, Wiltshire, UK) and confirmed to be authentic and mycoplasma-free. Het-1A, a normal esophageal cell line, was cultured in BEGM BulletKit medium without GA-1000 (Lonza Bioscience, Walkersville, MD, USA) and were cultured on T-75 tissue culture flasks pre-coated with 0.01 mg/mL fibronectin, 0.03 mg/mL bovine collagen type I, and 0.01 mg/mL bovine serum albumin (all from ThermoFisher Scientific) dissolved in the medium. Het-1A was acquired from the American Type Culture Collection (ATCC, Manassas, VA, USA) and confirmed to be authentic mycoplasma-free.

### siRNA knockdown of *TTLL12* and *HM13* isoforms and in vitro assays

*TTLL12* and *HM13* specific isoforms were selected for knockdown studies because both are among the top isoforms significantly linked with patient mortality. In addition, IS of both isoforms was validated via qRT-PCR, and it is feasible to design isoform-specific siRNA for these isoforms. Isoform-specific siRNAs of *TTLL12* were synthesized by MilliporeSigma (Burlington, MA, USA) using previously published sequences, and pre-designed isoform-specific siRNAs of *HM13* (SASI_Hs01_00100621 and SASI_Hs01_00100625) were acquired from MilliporeSigma [41]. Specificity of siRNA used in this study was confirmed using the NCBI BLAST [73]. Twenty-four hours prior to transfection, OE19 (140 000 cells/well), OE33 (4 000 cells/well), and Het-1A (200 000 cells/well) cells were seeded in black-walled, clear-bottom 96-well plates. On the day of transfection, Lipofectamine RNAiMAX Reagent (ThermoFisher Scientific) prepared in Opti-MEM medium was combined with 100 nM of siRNA prepared in Opti-MEM medium (ThermoFisher Scientific), followed by 20 minutes of incubation. Then, the siRNA-lipid complex was added to both cell lines and incubated for 72 h. Next, OE19 and OE33 cells were treated with either combination chemo drugs (1 μM paclitaxel and 5 μM carboplatin) or vehicle control (0.01% water and 0.1% DMSO; *n* = 6/condition). Both treatments and vehicle were prepared in RPMI 1640 medium with 2.0 mM L-glutamine, 10^4^ units/mL penicillin, 10^4^ μg/mL streptomycin, 1 mM sodium pyruvate, and 5% FBS. OE19 and OE33 EAC cells were stained using Calcein-AM (ThermoFisher Scientific) for in vitro viability measures at 24, 48, and 72 h post-treatment, as previously reported [15]. Het-1A normal esophageal cells were stained using Calcein-AM at 48 and 72 h post-siRNA-knockdown. Fluorescent images and readings were acquired using the SpectraMax MiniMax Imaging Cytometer (Molecular Devices, San Jose, CA, USA). Synergy was determined using CompuSyn (http://www.combosyn.com/).

### In vitro migration assay of OE19 and OE33 EAC cells treated with siRNA

To assess alteration in migration, OE19 and OE33 cells were seeded in T-25 tissue culture flasks at 3E6 cells/flask and 1.5E6 cells/flask, respectively. Twenty-four hours after seeding, both cell lines were similarly transfected with siRNA and incubated for 48 h. Then, cells were trypsinized with TrypLE Express (ThermoFisher Scientific) and seeded in 12-well plates at 1.5E6 cells/well and 1.3E6 cells/well, respectively. Following an overnight incubation, transfected cells were similarly treated with either combination chemo drugs or vehicle as described above. At 24 h post-treatment, cells reached about 90–100% confluency, and a vertical scratch was created in each well using a P-200 pipette tip. Microscope images were taken immediately after creating the scratch (*n* = 7 images per well). Scratch closure was measured every 24 h for up to 96 h with microscopic images of the same fields initially scratched measured. Distance of the scratch was quantified using Fiji software with the wound healing size tool [74, 75].

### In vitro antibody-dependent cell-mediated cytotoxicity (ADCC) assay

To measure whether isoform knockdown increases response to immunotherapy agents in EAC cells, the ADCC assay was performed using the ADCC Reporter Bioassay (Promega, Madison, WI, USA) following the manufacturer’s instructions. OE19 and OE33 cells were seeded in T-25 tissue culture flasks at 3E6 cells/flask and 1.5E6 cells/flask, respectively, and transfected with siRNA (NTC or pooled *HM13* siRNA) and incubated for 72 h. Then, OE19 and OE33 cells were trypsinized and seeded in white flat-bottom 96-well plates at 25 000 cells/well and 12 500 cells/well, respectively. Following an overnight incubation, OE19 and OE33 cells were treated with either avelumab, an anti-PD-L1 agent (MedChemExpress, Monmouth Junction, NJ, USA), or Human IgG1 Isotype Control (ThermoFisher Scientific) at 2, 4, and 8 μg/mL (*n* = 3/treatment). Treatment concentrations of avelumab were determined based on previously published studies [76, 77]. Immediately following treatment, ADCC Bioassay Effector Cells were added to each well at 75 000 cells/well and incubated for 6 h. The effector-to-target cell (E:T) ratio was 3:1 and 6:1 for OE19 and OE33 cells, respectively. Bio-Glo Luciferase Assay Reagent was then added to each well, and luminescence was measured using the SpectraMax MiniMax Imaging Cytometer (Molecular Devices). Fold change of induction was calculated by dividing the Relative Light Unit (RLU) from each treated well with RLU from non-antibody control.

### In vitro viability assay of OE19 and OE33 cells treated with thapsigarin, tunicamycin, and APR-246

OE19 and OE33 cells were seeded in black-walled, clear-bottom 96-well plates at 150 000 cells/well and 8 000 cells/well, respectively. Followed by overnight incubation, both cells were treated with thapsigarin (1 μM and 2 μM; MilliporeSigma), tunicamycin (50 ng/mL, 250 ng/mL, and 500 ng/mL; MilliporeSigma), APR-246 (30 μM and 60 μM; Cayman Chemical, Ann Arbor, MI, USA), or Vehicle (0.1% DMSO; *n* = 6/condition). Both treatments and vehicle were prepared in RPMI 1640 medium with 2.0 mM L-glutamine, 10^4^ units/mL penicillin, 10^4^ μg/mL streptomycin, 1 mM sodium pyruvate, and 5% FBS. Concentrations of thapsigarin, tunicamycin, and APR-246 treatment were determined from previously published studies [11, 22, 78]. In vitro cell viability was similarly measured using Calcein-AM at 24 and 48 h post-treatment. Fluorescent images and readings were acquired using the SpectraMax MiniMax Imaging Cytometer. Cell lysates were harvested using RPPA lysis buffer after viability measurement, as previously described [79].

### Lysate collection, SUnSET assay, and immunoblotting

Both OE19 and OE33 cells were seeded in T25 tissue culture flasks, transfected with siRNA, and treated with chemo drugs, as described above. Cell lysates were harvested using RPPA lysis buffer. To measure changes in global protein translation, the SUnSET assay was performed. Cell lysates for the SUnSET assay were treated with 1 μM puromycin for 30 min prior to lysate collection [80]. Protein was quantified using the DC Protein Assay (Bio-Rad, Hercules, CA, USA). Immunoblotting was performed using precast Criterion TGX Gels (Bio-Rad) with commercially available antibodies from Abcam (Waltham, MA, USA), Novus Biologicals (Littleton, CO, USA), Cell Signaling Technology (Danvers, MA, USA), ThermoFisher Scientific, MilliporeSigma, Santa Cruz Biotechnology (Dallas, TX, USA), and Developmental Studies Hybridoma Bank (Iowa City, IA, USA) (Table S2). Protein expression was quantified using ImageLab software (Bio-Rad) and normalized to loading controls (GAPDH or HSP60). siRNA transfection efficiency was also validated using protein detection via Western blots. Full-length, uncropped original Western blots are available as a Supplementary File.

### Statistical analysis

For cell-based experiments, all treatment groups had a similar confluency at experimental initiation to ensure unbiased results following treatments. Statistical analysis of in vitro viability, migration, and ADCC assays was performed using Prism (version 10.0.3, GraphPad Software, San Diego, CA, USA) via one-way ANOVA with Tukey’s post-hoc test. Isoform switches linked with patient survival were identified using the Cox proportional-hazards model via the Survival package (version 3.5-5) in R. *P*-values ≤ 0.05 were considered statistically significant.

## Supplementary information

Supplementary Figures_Tables and Methods 11.21.25

Western blots, Full length, Uncropped, Original blots

## Data Availability

RNA-seq data analyzed in this study are available in the NCBI Gene Expression Omnibus (GEO: GSE193946).
