# Supplementary material for: Identification of isoform switching events linked with esophageal adenocarcinoma patient survival informs novel prognostic and therapeutic targets
Source: Cell Death Dis. 2026 Mar 11;17(1):305. doi: 10.1038/s41419-026-08542-2 (PMC13039347; doi:10.1038/s41419-026-08542-2)
Supplement: Supplementary file 1 — Supplementary Figures_Tables and Methods 11.21.25 [file 41419_2026_8542_MOESM1_ESM.pdf]

Supplementary Methods

Rat reflux-induced EAC model

Reflux-induced EAC was created by performing esophagogastroduodenal anastomosis (EGDA) on 5 to 7-week-old male Sprague-Dawley rats, as we previously published (1). Animals were randomized to treatment groups based on mean body weights, ensuring there were no statistically significant differences at study initiation in body weights among the study groups. The EGDA procedure creates chronic reflux of bile and acidic gastric contents into the lower esophagus, mimicking human gastroesophageal reflux disease (GERD) or reflux-driven Barrett's esophagus, the only known precursor lesion to EAC. Animals were sacrificed at 40 weeks after EGDA surgery. Organs were flash-frozen in liquid nitrogen and stored at -80 °C until further processing. All animal procedures were conducted in accordance with the protocol approved by the Institutional Laboratory Animal Care and Use Committee at the Medical College of Wisconsin (AUA3095). All animals that completed the study and confirmed to have a successful anastomosis were included in the analysis as previously detailed (1). The sample size per treatment group was based on the previously conducted chemoprevention study and was based on sufficient sample size required to determine a statistically significant difference between the EGDA positive group and chemoprevention treated group at 40 weeks of study and to measure the study outcomes (i.e., metabolomics, proteomics, transcriptional profiles, tumorgenicity measures, serology measures, and microbiome profiles) as previously detailed (1).

RNA-sequencing of rat esophageal tissue and analysis

RNA was isolated from flash-frozen esophagi harvested from rats in two treatment groups (Water or negative controls and Reflux or positive EAC controls) using homogenization and the Qiagen RNeasy Fibrous Tissue Kit (Germantown, Maryland, USA). RNA concentration and integrity were measured by Nanodrop and Agilent Nanochip (Santa Clara, CA, USA) analyses, respectively. Prior to sample submission, the concentration of each sample was adjusted to 30 ng/µL and 20 µL per sample was submitted to BGI Americas for RNA-sequencing (RNA-seq). All samples were confirmed to be of sufficient quantity and integrity by BGI Americas (Cambridge, MA, USA) prior to beginning library construction. The library was prepared with 100 bp paired-end reads using the Illumina HiSeq 4000 sequencing platform (San Diego, CA, USA). Quality control was performed on the raw sequencing reads using FastQC (2). Then, isoform quantification was performed using kallisto (version 0.46.0) (3), followed by differential isoform and gene expression analyses using sleuth (version 0.30.2) in R (version 4.5.2) (4). Kallisto transcriptome indices were constructed from Ensembl rat reference transcriptomes (version 96) (5).

Transcriptomics analysis of TCGA data

Transcriptomic analysis of TCGA data was performed using computational resources and services provided by Advanced Research Computing at the University of Michigan, Ann Arbor. Controlled-access TCGA data were requested through the Database of Genotypes and Phenotypes (dbGAP, accession number: phs000178). Primary sequence data (BAM files) of EAC samples (n = 88) and normal esophageal tissues (n = 9) were identified and downloaded from the NCI Genomic Data Commons (GDC) (6). Then, FASTQ files were extracted from downloaded BAM files using the samtools

(version 1.21) (7), followed by isoform quantification using kallisto (version 0.46.0) (3). Kallisto transcriptome indices were constructed from the Ensembl human reference transcriptomes (version 96) (5). Differential isoform expression analysis was performed using sleuth (version 0.30.2) in R (version 4.5.2) (4).

Quantification of gene and isoform expression of siRNA-treated EAC cells using qPCR

qPCR analysis of siRNA-treated EAC cells was performed using the Cells-to-Ct 1-Step PowerSYBR Green Kit (ThermoFisher Scientific), following the manufacturer's instructions. In short, OE19 and OE33 cells were seeded at 6 000 cells/well in 12-well plates. Following an overnight incubation, EAC cells were treated with 100nM of siRNA, prepared using Lipofectamine RNAiMAX Reagent (ThermoFisher Scientific) and Opti-MEM medium (ThermoFisher Scientific). At 72h post-transfection, cell lysates were collected using DNase/Lysis Solution. Then, 1-step RT-PCR was performed using QuantStudio 3 Real-Time PCR (ThermoFisher Scientific). Relative isoform and gene expression was determined using *GAPDH* as the housekeeping gene and normalized against non-targeting control (NTC)-treated cells, using the $\Delta\Delta$Cq calculation method. Sequences of primers used in qPCR are reported in Table S1.

Table S1. qRT-PCR primer sequences

Gene	Isoform	Primer sequence (5' to 3')
ATP9B	207	Forward: GACCAGATCCCGCTTTACCC<br>Reverse: GCATTATCCTGGCTCGTGGT
	208	Forward: GTGACTCTGCAAATAATGCCCT<br>Reverse: AGGCCAGCTCCACAAATTCAT
HM13	201	Forward: GGAGGGAGCACGTCACTTC<br>Reverse: CACTGCCGTTATGCGGATCG
	221	Forward: GCCACTGACTTGCTATGGTC<br>Reverse: TCTGTCACTTCTCCCTTGGC
	multiple	Forward: TGTTGCCTTAGGGGAACGTG<br>Reverse: ATCCCGGCTGGTGATTGTTT
KIF16B	201	Forward: ATCACACCTGGTTCCCCTCAT<br>Reverse: GGGTCCTTCAGGTCATCTGGATTT
	203	Forward: CCTGATGCCAGAGCCTGATG<br>Reverse: CAGAAGGACTAGCGACTGGC
	205	Forward: AGAGATGCCACTGACACTTCC<br>Reverse: TTTGGTTCAAAATGGCCAGCAT
TTLL12	201	Forward: TCATGGACGAGTTCGGTTCG<br>Reverse: TCACTCTGGGTGAGGGTGAA
	204	Forward: GTTCCTGCTATGGAAGGCGA<br>Reverse: AAGCACAGGTTTTGGGGACA
	multiple	Forward: CTGAGATCTTCCGGGCCTTC<br>Reverse: CATGAGGTCGACGGCATACA

Table S2. Immunoblot primary antibody information.

Primary Antibody	Antibody Company	Catalog Number	Dilution Factor
ATF-4	Cell Signaling Technology	11815	1:1000
ATF-6	Cell Signaling Technology	65880	1:1000
BAK	Cell Signaling Technology	12105	1:1000
BiP	Cell Signaling Technology	3177	1:1000
Chk1	Cell Signaling Technology	2360	1:1000
GADD34	Invitrogen	PA1-139	1:1000
GAPDH	Cell Signaling Technology	2118	1:25000
GAPDH	Santa Cruz	sc-32233	1:30000
HM13	Abcam	ab247061	1:500
HSC70	Invitrogen	PA5-27337	1:1000
HSP60	Cell Signaling Technology	12165	1:7500
IRE-1α	Cell Signaling Technology	3294	1:1000
LAMP2A	Abcam	18528	1:1000
LC3	Cell Signaling Technology	4108	1:1000
NDP52	Abcam	68588	1:1000
p-Chk1	Cell Signaling Technology	2348	1:1000
p-eIF2α	Cell Signaling Technology	3597	1:1000
PARP	Cell Signaling Technology	9532	1:1000
PERK	Cell Signaling Technology	5683	1:1000
PINK1	Cell Signaling Technology	6946	1:1000
p-JNK	Cell Signaling Technology	4668	1:1000
Puromycin	Developmental Studies Hybridoma Bank	PMY-2A4	1:1000
TTLL12	Novus Biologicals	NBP2-02216	1:1000
TP53	Millipore Sigma	OP43	1:1000
XBP-1	Cell Signaling Technology	40435	1:1000

REFERENCES

1. Weh KM, Howard CL, Zhang Y, Tripp BA, Clarke JL, Howell AB, et al. Prebiotic proanthocyanidins inhibit bile reflux-induced esophageal adenocarcinoma through reshaping the gut microbiome and esophageal metabolome. JCI Insight. 2024;9(6).
2. FastQC. 2015.
3. Bray NL, Pimentel H, Melsted P, Pachter L. Near-optimal probabilistic RNA-seq quantification. Nat Biotechnol. 2016;34(5):525–7.
4. Pimentel H, Bray NL, Puente S, Melsted P, Pachter L. Differential analysis of RNA-seq incorporating quantification uncertainty. Nat Methods. 2017;14(7):687–90.
5. Dyer SC, Austine-Orimoloye O, Azov AG, Barba M, Barnes I, Barrera-Enriquez VP, et al. Ensembl 2025. Nucleic Acids Res. 2025;53(D1):D948–D57.
6. Heath AP, Ferretti V, Agrawal S, An M, Angelakos JC, Arya R, et al. The NCI Genomic Data Commons. Nat Genet. 2021;53(3):257–62.
7. Li H, Handsaker B, Wysoker A, Fennell T, Ruan J, Homer N, et al. The Sequence Alignment/Map format and SAMtools. Bioinformatics. 2009;25(16):2078–9.

A

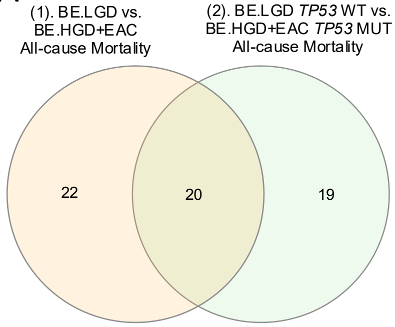

Unique to (1):
COL4A2 ENST00000360467
COL4A2 ENST00000463084
IGSF3 ENST00000369483
IGSF3 ENST00000369486
RAB30 ENST00000527633
RAB30 ENST00000534301
TTLL12 ENST00000216129
TTLL12 ENST00000494035
UGT1A1 ENST00000305208
UGT1A1 ENST00000360418
PCSK5 ENST00000376752
PCSK5 ENST00000674117
RNF128 ENST00000255499
RNF128 ENST00000324342
MEP1B ENST00000269202
MEP1B ENST00000581184
MINDY1 ENST00000470877
MINDY1 ENST00000361936
RNFT1-DT ENST00000593015
RNFT1-DT ENST00000586209
ESPN ENST00000636644
ESPN ENST00000475479

Unique to (2):
HM13 ENST00000493364
HM13 ENST00000340852
GPR4 ENST00000591614
MPZ ENST00000488271
RPL22L1 ENST00000475836
RPL22L1 ENST00000463836
CLCA4 ENST00000370563
DANT2 ENST00000430756
DANT2 ENST00000664164
GPR4 ENST00000323040
KRAS ENST00000311936
KRAS ENST00000256078
MPZ ENST00000533357
MPZ ENST00000672602
MIR223HG ENST00000618234
MIR223HG ENST00000621933
CLCA4 ENST00000496322
MIR4458HG ENST00000502001
MIR4458HG ENST00000652260

Shared:
KIF16B ENST00000635823
FSIP2 ENST00000415915
CFDP1 ENST00000570103
CFDP1 ENST00000283882
TSPAN6 ENST00000494424
TSPAN6 ENST00000614008
FSIP1 ENST00000559547
TPM4 ENST00000646974
TPM4 ENST00000653979
FSIP1 ENST00000350221
KIF16B ENST00000354981
SMIM6 ENST00000556126
SMIM6 ENST00000579469
AC021752.1 ENST00000613161
AC021752.1 ENST00000656830
FSIP2 ENST00000429412
C1orf54 ENST00000369098
C1orf54 ENST00000369102
ARL10 ENST00000310389
ARL10 ENST00000514533

B

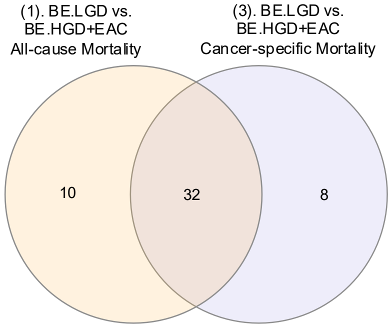

Unique to (1):
COL4A2 ENST00000360467
COL4A2 ENST00000463084
TTLL12 ENST00000216129
TTLL12 ENST00000494035
RNF128 ENST00000255499
RNF128 ENST00000324342
C1orf54 ENST00000369098
C1orf54 ENST00000369102
MEP1B ENST00000269202
MEP1B ENST00000581184

Unique to (3):
KRAS ENST00000311936
KRAS ENST00000256078
SLC28A3 ENST00000376238
SLC28A3 ENST00000495823
MSH5-SAPCD1 ENST00000493662
MSH5-SAPCD1 ENST00000476085
FSIP2-AS1 ENST00000429929
FSIP2-AS1 ENST00000436557

Shared:
KIF16B ENST00000635823
FSIP2 ENST00000415915
CFDP1 ENST00000570103
CFDP1 ENST00000283882
TSPAN6 ENST00000494424
TSPAN6 ENST00000614008
FSIP1 ENST00000559547
TPM4 ENST00000646974
TPM4 ENST00000653979
IGSF3 ENST00000369483
IGSF3 ENST00000369486
RAB30 ENST00000527633
RAB30 ENST00000534301
FSIP1 ENST00000350221
KIF16B ENST00000354981
SMIM6 ENST00000556126
SMIM6 ENST00000579469
UGT1A1 ENST00000305208
UGT1A1 ENST00000360418
PCSK5 ENST00000376752
PCSK5 ENST00000674117
AC021752.1 ENST00000613161
AC021752.1 ENST00000656830
FSIP2 ENST00000429412
ARL10 ENST00000310389
ARL10 ENST00000514533
MINDY1 ENST00000470877
MINDY1 ENST00000361936
RNFT1-DT ENST00000593015
RNFT1-DT ENST00000586209
ESPN ENST00000636644
ESPN ENST00000475479

C

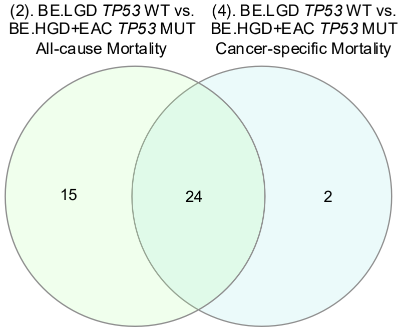

Unique to (2):
TSPAN6 ENST00000614008
TSPAN6 ENST00000494424
MPZ ENST00000488271
RPL22L1 ENST00000475836
RPL22L1 ENST00000463836
ARL10 ENST00000310389
ARL10 ENST00000514533
MPZ ENST00000533357
MPZ ENST00000672602
MIR223HG ENST00000618234
MIR223HG ENST00000621933
C1orf54 ENST00000369098
C1orf54 ENST00000369102
MIR4458HG ENST00000502001
MIR4458HG ENST00000652260

Unique to (4):
LINC02542 ENST00000660534
LINC02542 ENST00000668266

Shared:
HM13 ENST00000493364
HM13 ENST00000340852
CFDP1 ENST00000570103
CFDP1 ENST00000283882
FSIP2 ENST00000415915
TPM4 ENST00000646974
TPM4 ENST00000653979
FSIP1 ENST00000559547
GPR4 ENST00000591614
SMIM6 ENST00000556126
SMIM6 ENST00000579469
CLCA4 ENST00000370563
DANT2 ENST00000430756
DANT2 ENST00000664164
AC021752.1
ENST00000613161
AC021752.1
ENST00000656830
FSIP2 ENST00000429412
KIF16B ENST00000354981
KIF16B ENST00000635823
GPR4 ENST00000323040
KRAS ENST00000311936
KRAS ENST00000256078
CLCA4 ENST00000496322
FSIP1 ENST00000350221

D

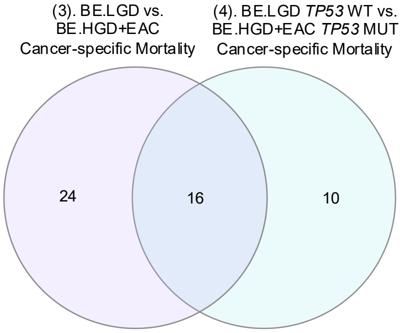

Unique to (3):
UGT1A1 ENST00000305208
UGT1A1 ENST00000360418
IGSF3 ENST00000369483
IGSF3 ENST00000369486
PCSK5 ENST00000376752
PCSK5 ENST00000674117
ESPN ENST00000636644
ESPN ENST00000475479
TSPAN6 ENST00000494424
TSPAN6 ENST00000614008
MINDY1 ENST00000470877
MINDY1 ENST00000361936
ARL10 ENST00000310389
ARL10 ENST00000514533
SLC28A3 ENST00000376238
SLC28A3 ENST00000495823
RNFT1-DT ENST00000593015
RNFT1-DT ENST00000586209
MSH5-SAPCD1 ENST00000493662
MSH5-SAPCD1 ENST00000476085
RAB30 ENST00000527633
RAB30 ENST00000534301
FSIP2-AS1 ENST00000429929
FSIP2-AS1 ENST00000436557

Unique to (4):
GPR4 ENST00000323040
GPR4 ENST00000591614
CLCA4 ENST00000496322
DANT2 ENST00000430756
DANT2 ENST00000664164
HM13 ENST00000493364
HM13 ENST00000340852
CLCA4 ENST00000370563
LINC02542 ENST00000660534
LINC02542 ENST00000668266

Shared:
AC021752.1 ENST00000613161
AC021752.1 ENST00000656830
FSIP2 ENST00000415915
KIF16B ENST00000635823
FSIP1 ENST00000559547
FSIP1 ENST00000350221
KIF16B ENST00000354981
TPM4 ENST00000646974
TPM4 ENST00000653979
FSIP2 ENST00000429412
KRAS ENST00000311936
KRAS ENST00000256078
CFDP1 ENST00000570103
CFDP1 ENST00000283882
SMIM6 ENST00000556126
SMIM6 ENST00000579469

Figure S1. Common and unique patient survival-linked isoforms among comparisons. (A-D) Venn diagram and the list of survival-linked isoforms shared or unique to each comparison. Each survival-linked isoform is listed by its gene name and Ensembl transcript ID. Positive and negative hazard ratios for significantly altered isoform fractions are detailed in Figure 2.

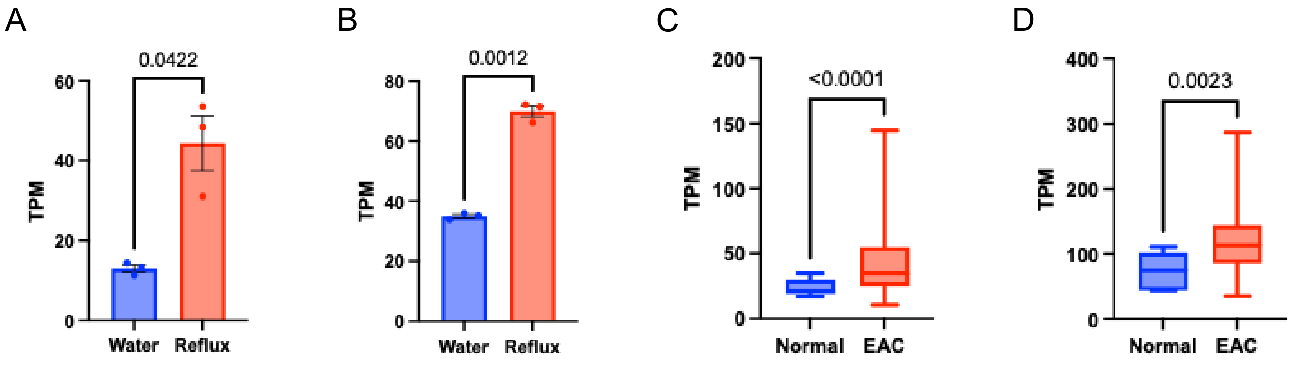

Figure S2. Expression of *TTLL12* and *HM13* in a reflux-induced EAC rat model and in EAC samples derived from a Cancer Genome Atlas Program (TCGA) cohort. Expression of (A) *Ttll12-201* (B) *HM13* ortholog, *Mcts2*, in rats with reflux-induced EAC (n=3) and water controls (n=3). Expression of (C) *TTLL12-201* and (D) *HM13-201* in TCGA EAC (n = 88) and normal (n = 9) tissue samples. TPM, transcripts per million.

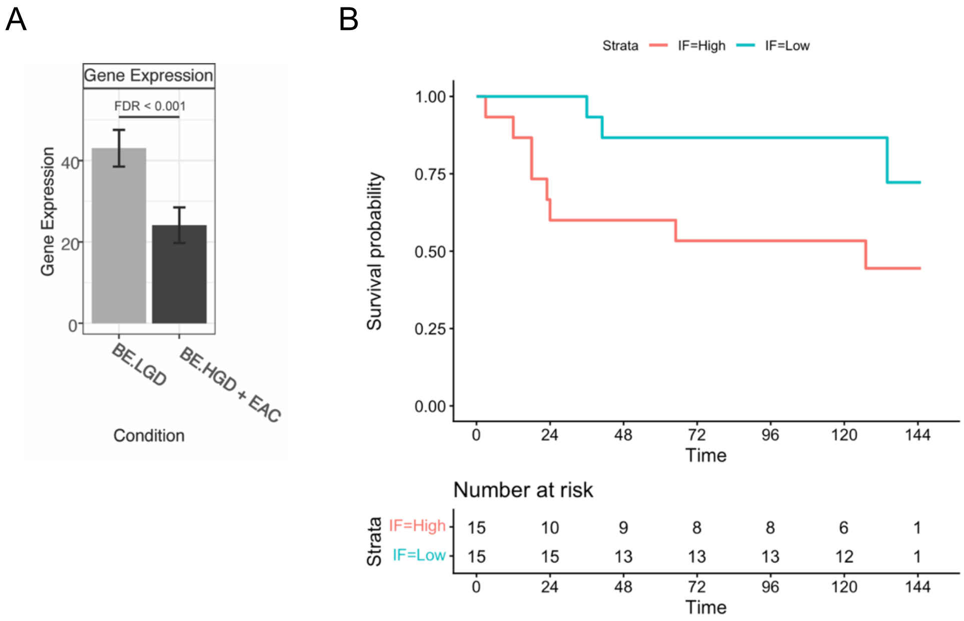

Figure S3. Isoform switching of *TTLL12* is linked with patient survival. (A) Bar plot showing the gene-level expression difference between BE.LGD and BE.HGD + EAC. (B) Survival plot of patients stratified based on isoform usage of *TTLL12-201*, with the risk table. FDR, false discovery rate; IF, isoform fraction.

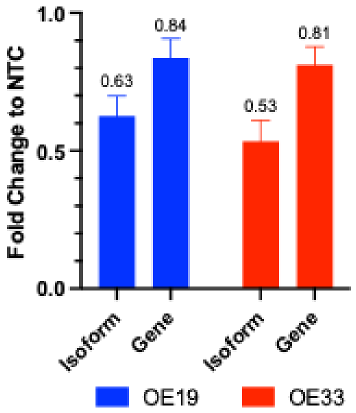

Figure S4. Expression of the *TTLL12-201* isoform and the *TTLL12* gene in human EAC cell lines following *TTLL12-201* siRNA knockdown. Expression levels were first normalized to GAPDH in each condition and then normalized to EAC cells treated with non-targeting control (NTC).

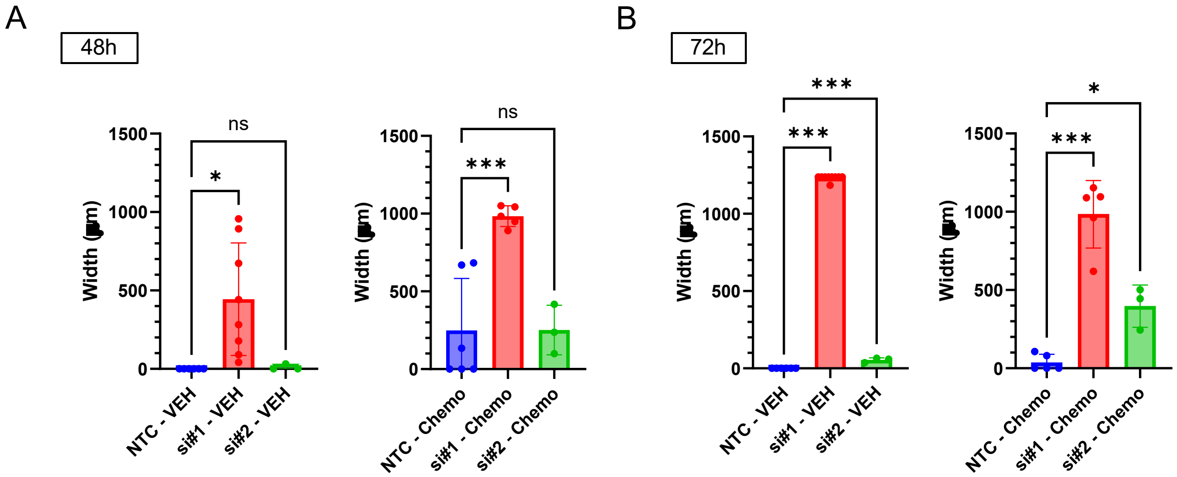

Figure S5. *TTLL12 i*soform-specific knockdown inhibits the migratory potential of OE33 cells. (A) Migration assay results of OE33 cells treated with siRNA alone or in combination with chemotherapy agents at 48h. (B) Migration assay results of OE33 cells treated with siRNA alone or in combination with chemotherapy agents at 72h. NTC, non-targeting control; ns, non-significant; *, $P < 0.05$; ***, $P < 0.001$.

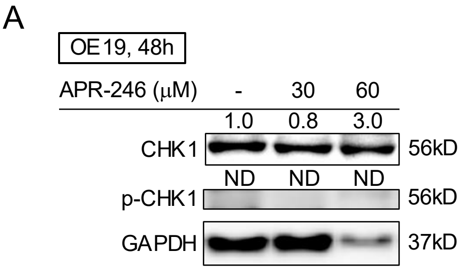

Figure S6. Immunoblots of OE19 and OE33 cells following APR-246 treatment. Immunoblots of CHK1 and phospho-CHK1 for (A) OE19 and (B) OE33 cells treated with APR-246 at 48h. ND, not detected.

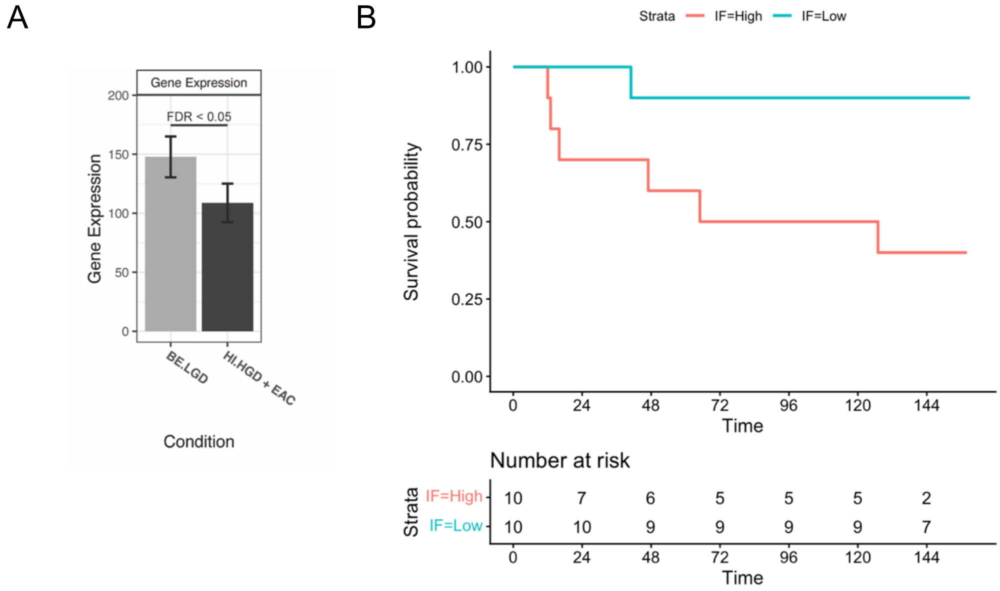

Figure S7. Isoform switching of *HM13* is linked with patient survival. (A) Bar plot showing the gene-level expression difference between BE.LGD and BE.HGD + EAC. (B) Survival plot of patients stratified based on isoform usage of *HM13-201*, with the risk table. FDR, false discovery rate; IF, isoform fraction.

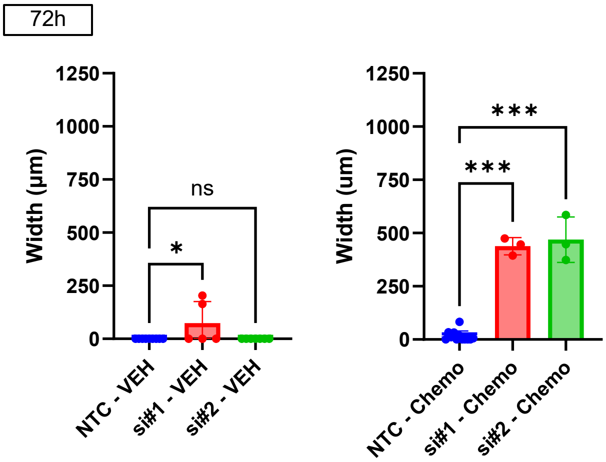

Figure S8. *HM13 i*soform-specific knockdown inhibits the migratory potential of OE33 cells at 72h. Bar plots showing the migration assay results of OE33 cells treated with siRNA alone or in combination with chemotherapy agents. NTC, non-targeting control; ns, non-significant; *, P < 0.05; ***, P < 0.001.

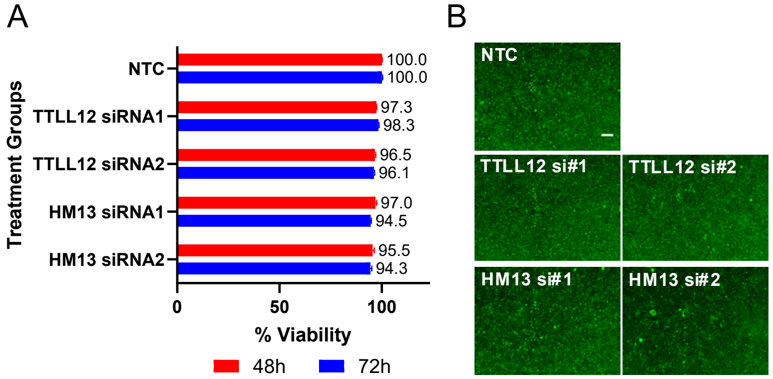

Figure S9. Isoform-specific knockdown of TTLL12 and HM13 in Het1A normal esophageal cells. (A) Viability results of Het1A cells treated with each siRNA (n=6/group). (B) Representative fluorescent images of Het1A cells treated with each siRNA at 48h. NTC, non-targeting control. Scale bar, 200 μm.
