# Supplementary material for: Identification of isoform switching events linked with esophageal adenocarcinoma patient survival informs novel prognostic and therapeutic targets
Source: Cell Death Dis. 2026 Mar 11;17(1):305. doi: 10.1038/s41419-026-08542-2 (PMC13039347; doi:10.1038/s41419-026-08542-2)

Identification of isoform switching events linked with esophageal adenocarcinoma patient survival informs novel prognostic and therapeutic targets

Yun Zhang, David A. Ntsiful, Rachel Israel, Bryce Vandenburg, Shari Barnett, Jean-Jack Riethoven, Jennifer L. Clarke, Kiran H. Lagisetty, Jules Lin, Rishindra M. Reddy, Andrew C. Chang, David D. Odell, Analisa DiFeo, Maureen A. Sartor, and Laura A. Kresty

<u>Original western blot images for data presented in the manuscript:</u>

Figure 3 – Panels D and E
Figure 4 – Panels A-D and F-G
Figure 5 – Panels D and E
Figure 6 – Panels A-B and D-E
Figure 7 – Panels C and D

Figure 3D-E.
TTLL12
Novus
Catalog #NBP2-02216
1:1000

GAPDH
Santa Cruz
Catalog #sc-32233
1:30000

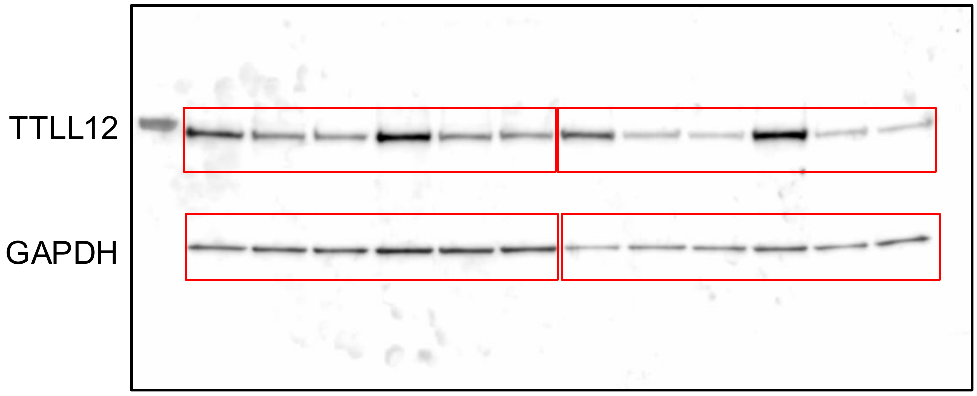

Figure 4A.
LAMP2A
Abcam
Catalog #18528
1:1000

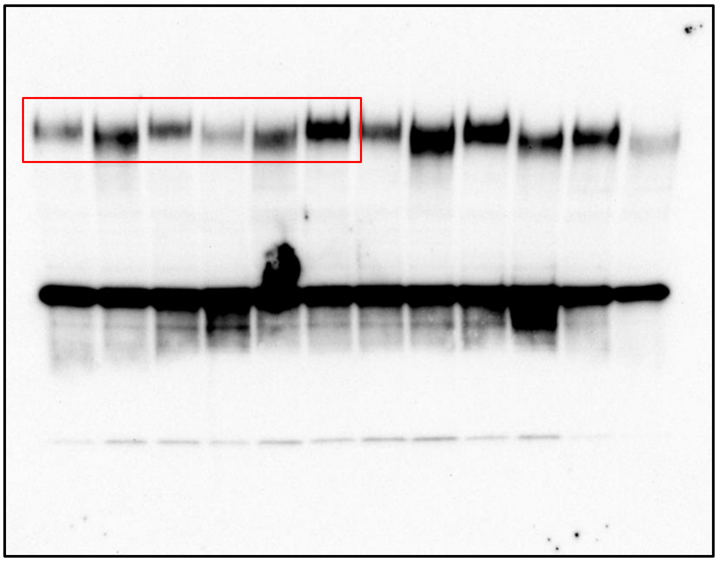

Figure 4A.
HSC70
Invitrogen
Catalog #PA5-27337
1:1000

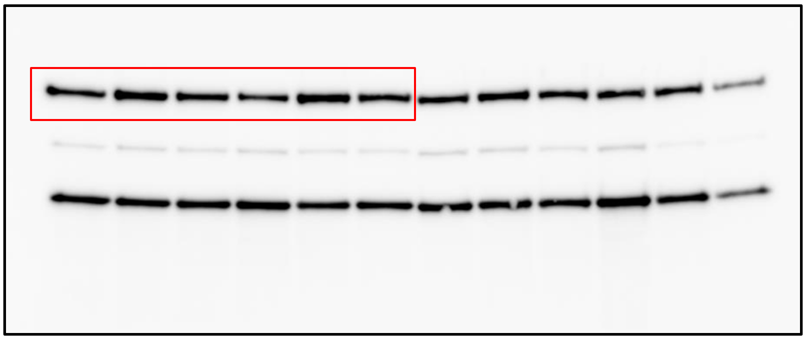

Figure 4A.
LC3
CST
Catalog #4108
1:1000

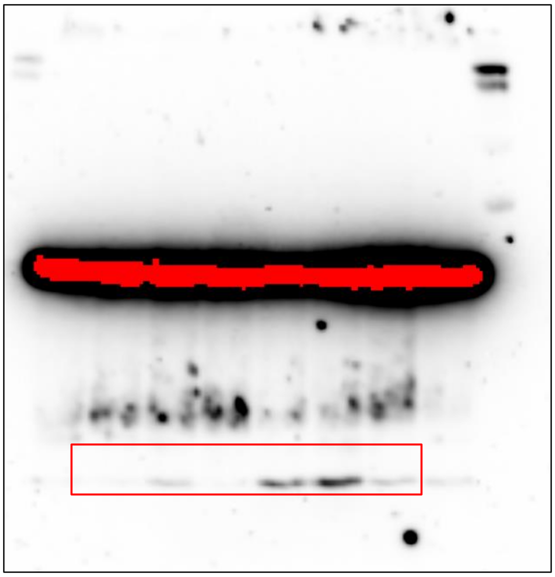

Figure 4A.
GAPDH
Cell Signaling
Catalog #2118
1:30000

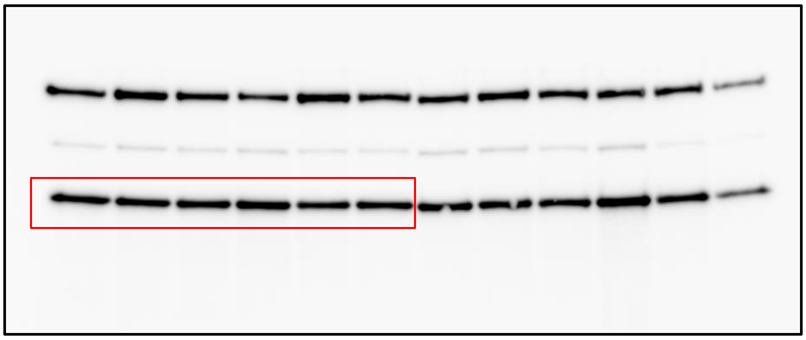

Figure 4B.
LAMP2A
Abcam
Catalog #18528
1:1000

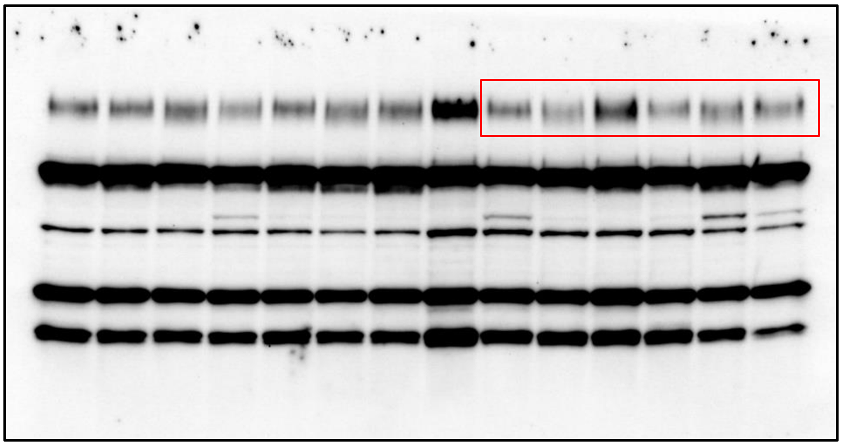

Figure 4B.
HSC70
Invitrogen
Catalog #PA5-27337
1:1000

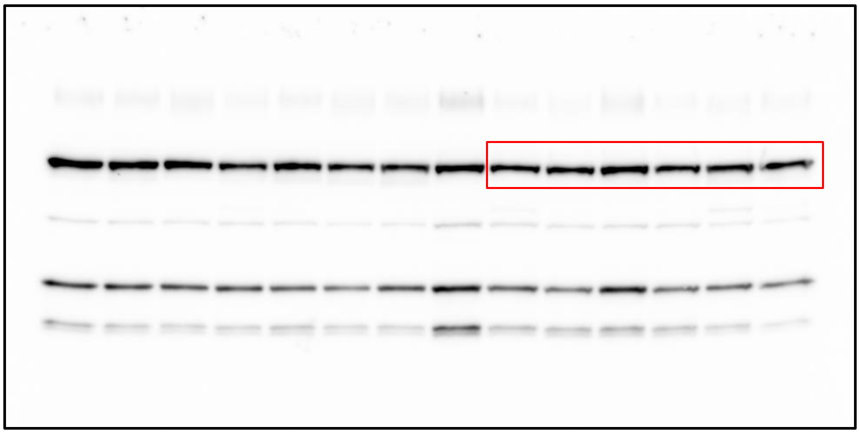

Figure 4B.
LC3
Cell Signaling
Catalog #4108
1:1000

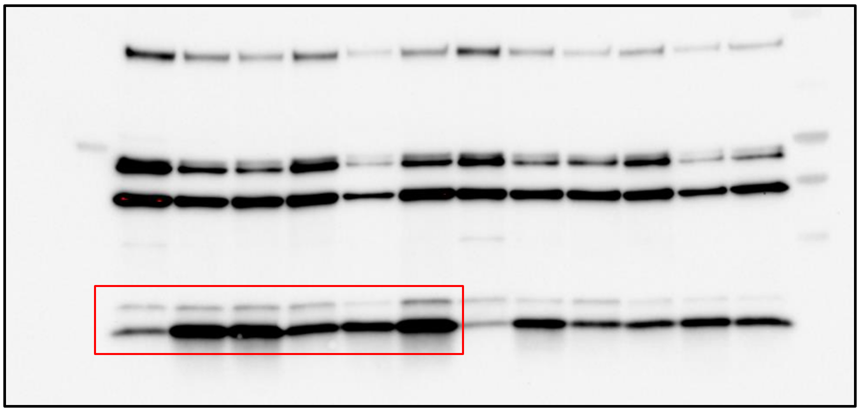

Figure 4B.
GAPDH
Cell Signaling
Catalog #2118
1:30000

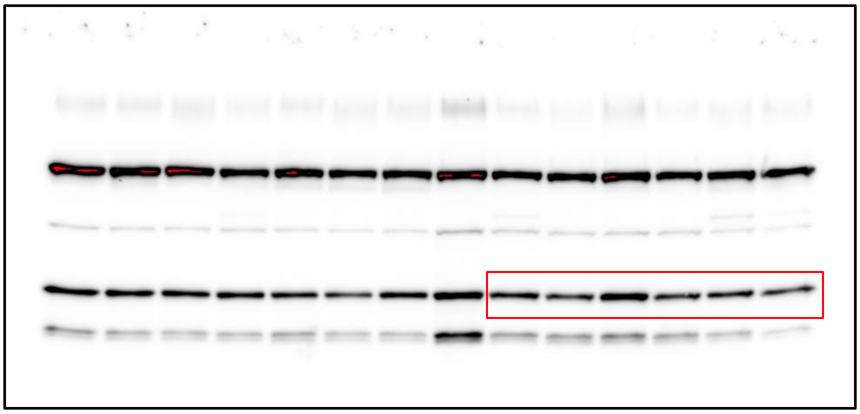

Figure 4C.
CHK1
Cell Signaling
Catalog #2360
1:1000

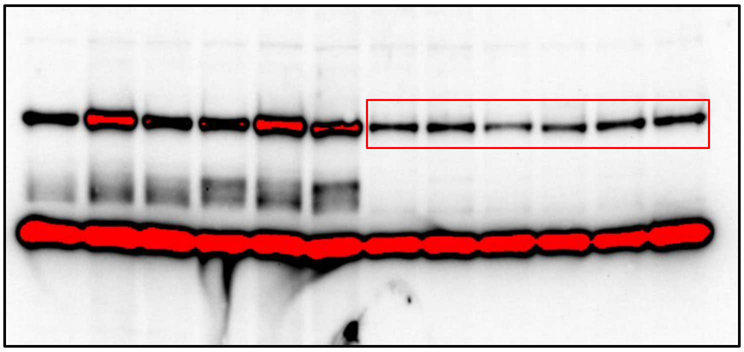

Figure 4C.
p-CHK1
Cell Signaling
Catalog #2348
1:1000

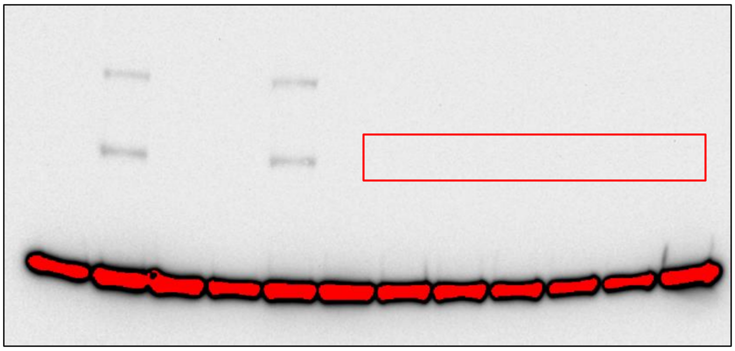

Figure 4C.
TP53
Millipore Sigma
Catalog #OP43
1:1000

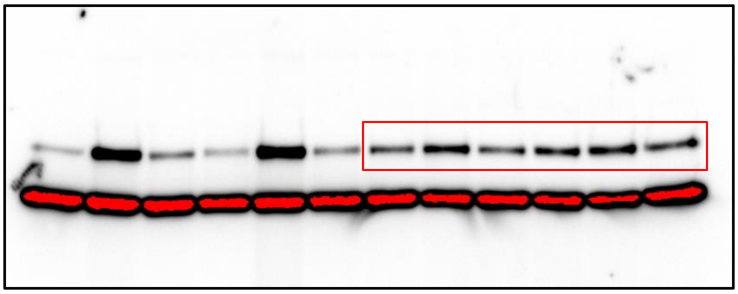

Figure 4C.
GAPDH
Santa Cruz
Catalog #sc-32233
1:25000

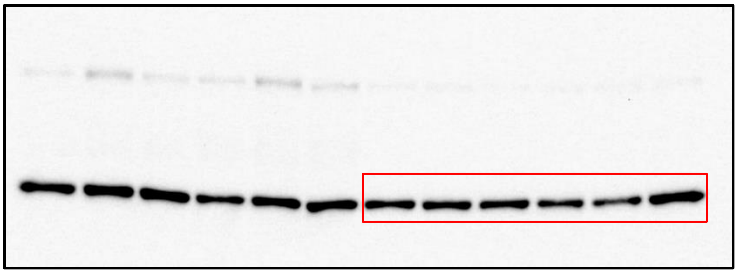

Figure 4D.
CHK1
Cell Signaling
Catalog #2360
1:1000

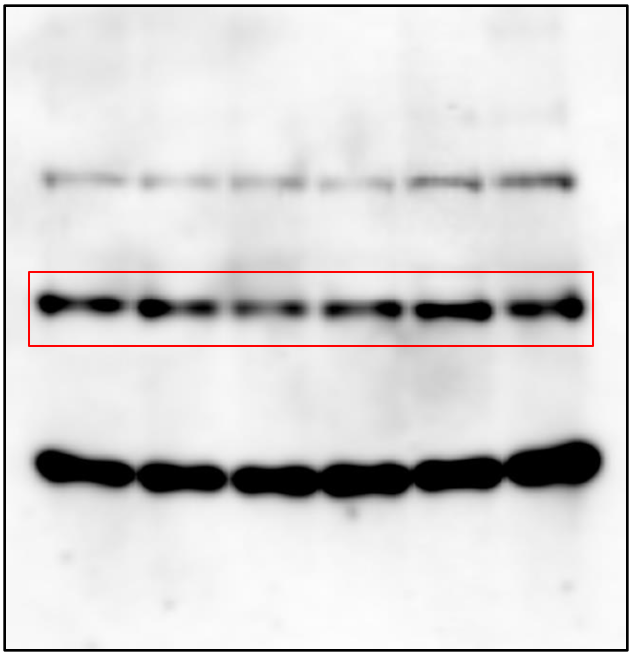

Figure 4D.
p-CHK1
Cell Signaling
Catalog #2348
1:1000

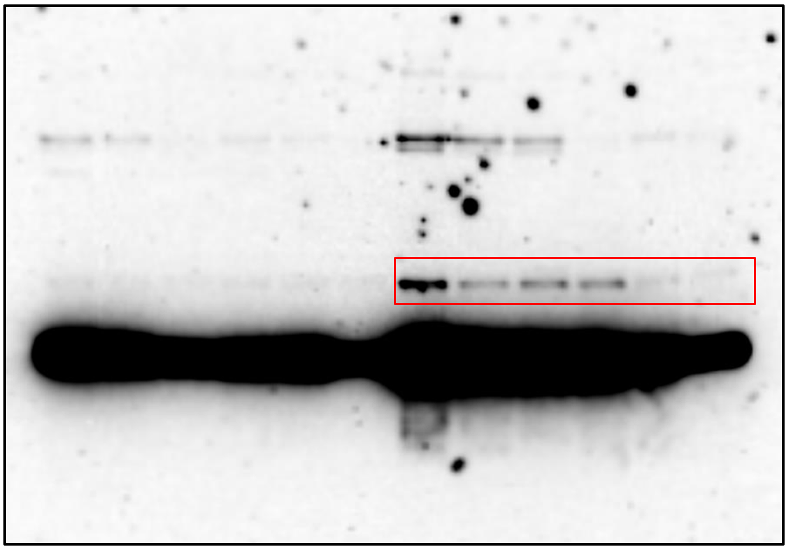

Figure 4D.
TP53
Milipore Sigma
Catalog #OP43
1:1000

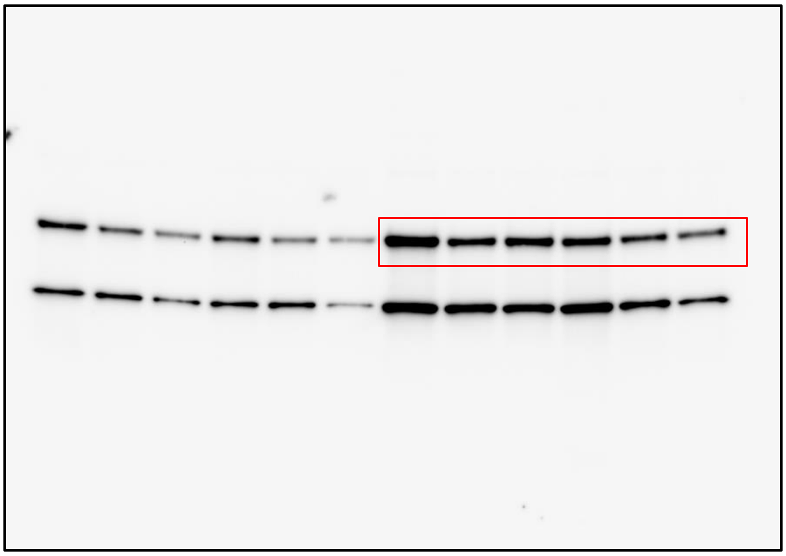

Figure 4D.
GAPDH
Santa Cruz
Catalog #sc-32233
1:25000

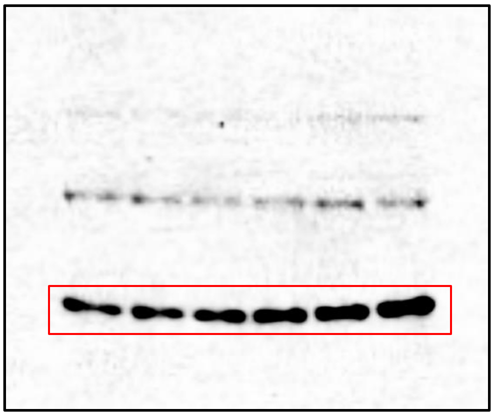

Figure 4F.
LAMP2A
Abcam
Catalog #18528
1:1000

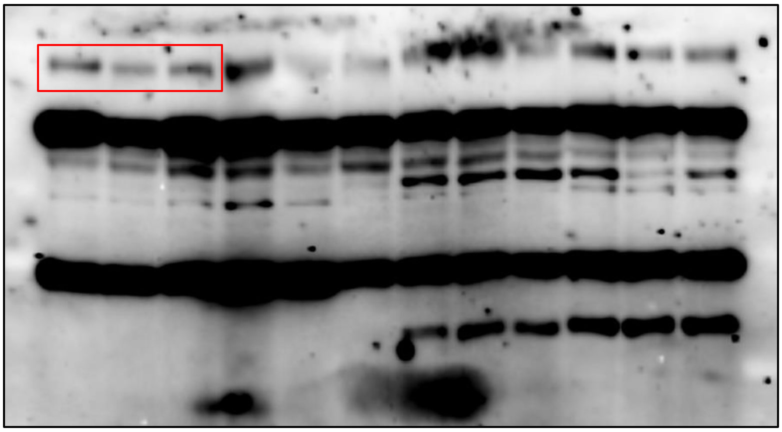

Figure 4F.
HSC70
Invitrogen
Catalog #PA5-27337
1:1000

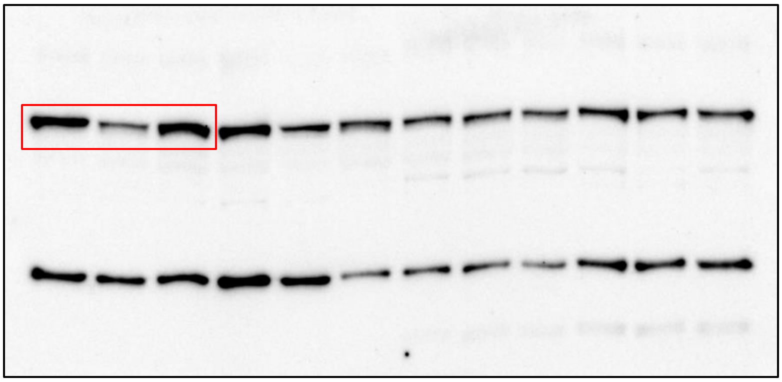

Figure 4F.
CHK1
Cell Signaling
Catalog #2360
1:1000

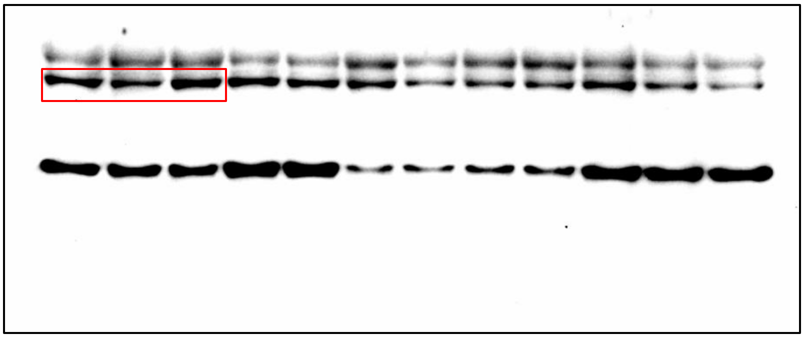

Figure 4F.
p-CHK1
Cell Signaling
Catalog #2348
1:1000

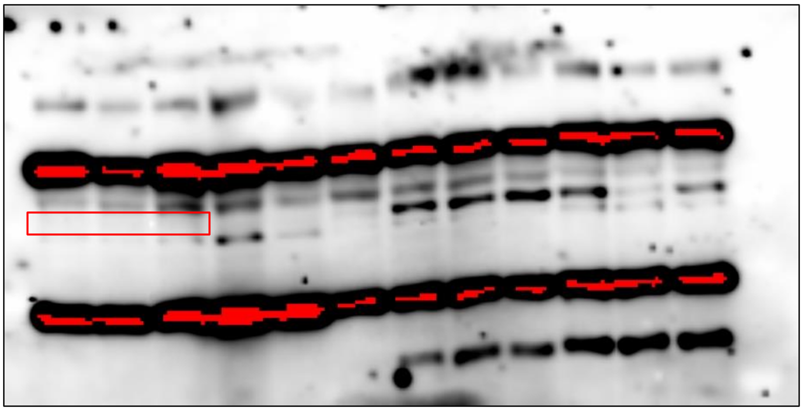

Figure 4F.
TP53
Millipore
Catalog #OP43
1:1000

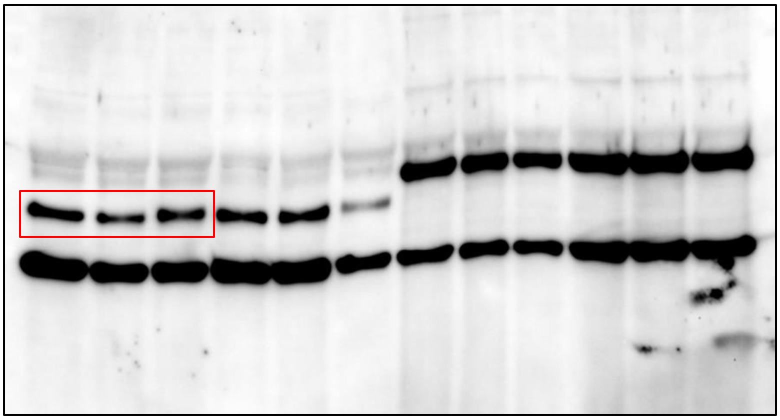

Figure 4F.
LC3
Cell Signaling
Catalog #4108
1:1000

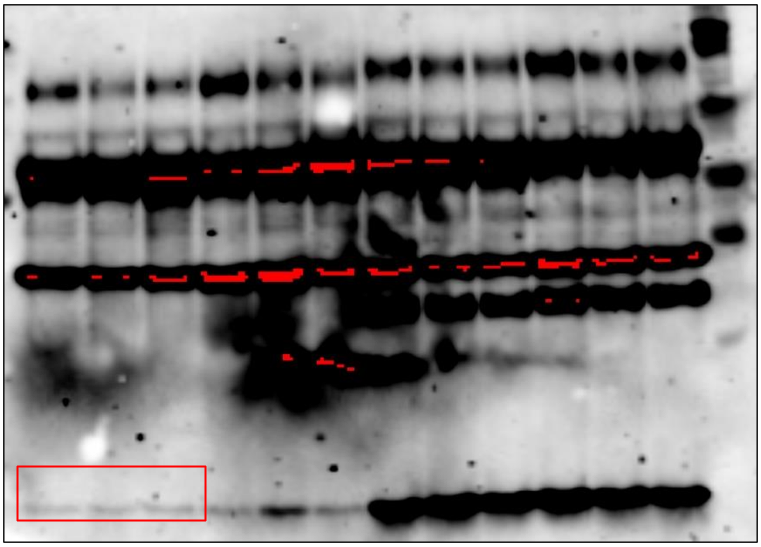

Figure 4F.
GAPDH
Cell Signaling
Catalog #2118
1:25,000

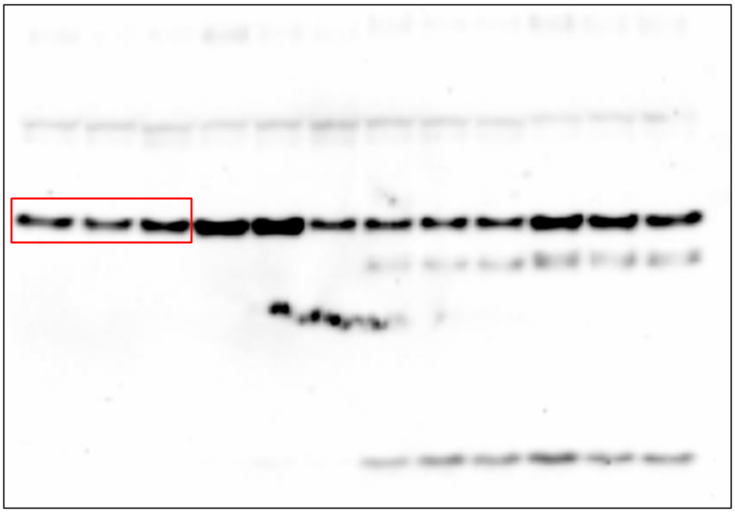

Figure 4G.
LAMP2A
Abcam
Catalog #18528
1:1000

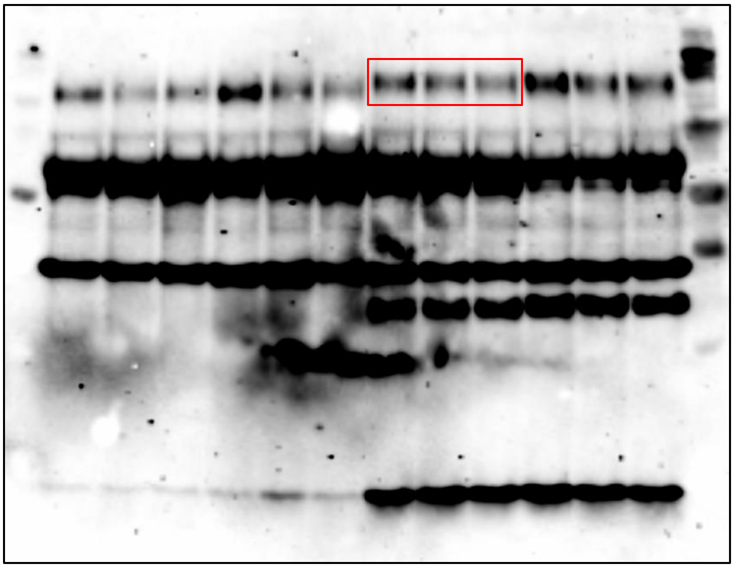

Figure 4G.
HSC70
Invitrogen
Catalog #PA5-27337
1:1000

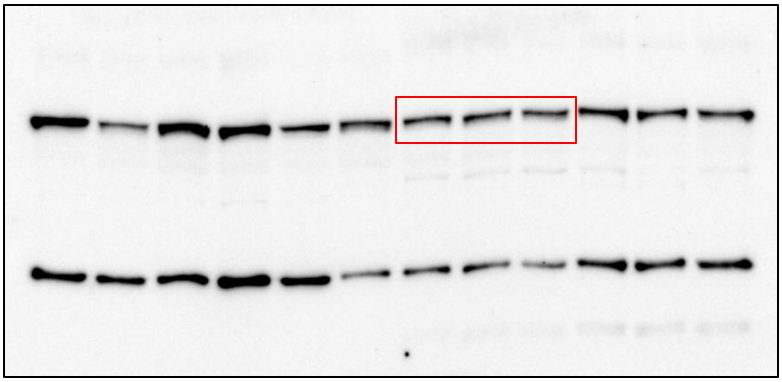

Figure 4G.
CHK1
Cell Signaling
Catalog #2360
1:1000

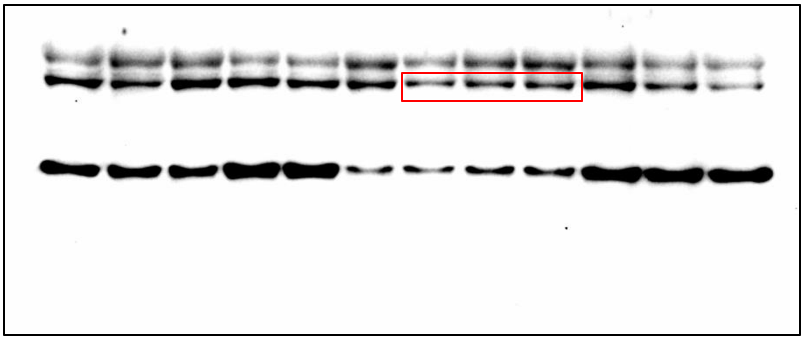

Figure 4G.
p-CHK1
Cell Signaling
Catalog #2348
1:1000

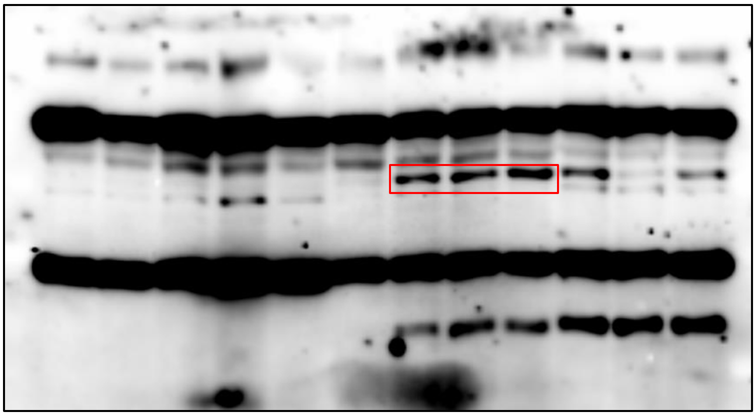

Figure 4G.
TP53
Millipore
Catalog #OP43
1:1000

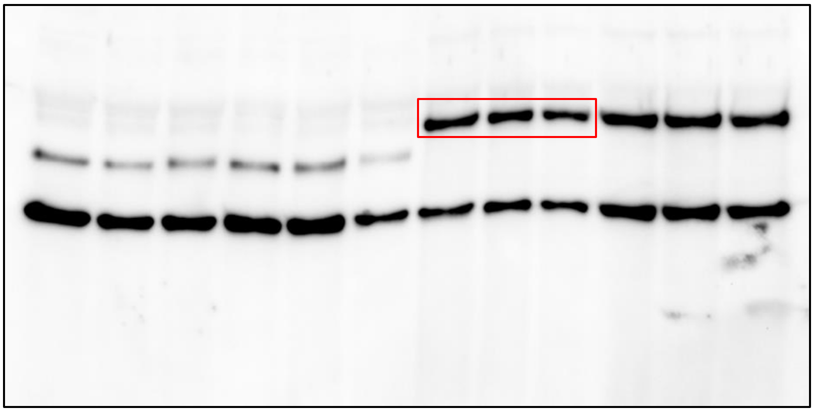

Figure 4G.
LC3
Cell Signaling
Catalog #4108
1:1000

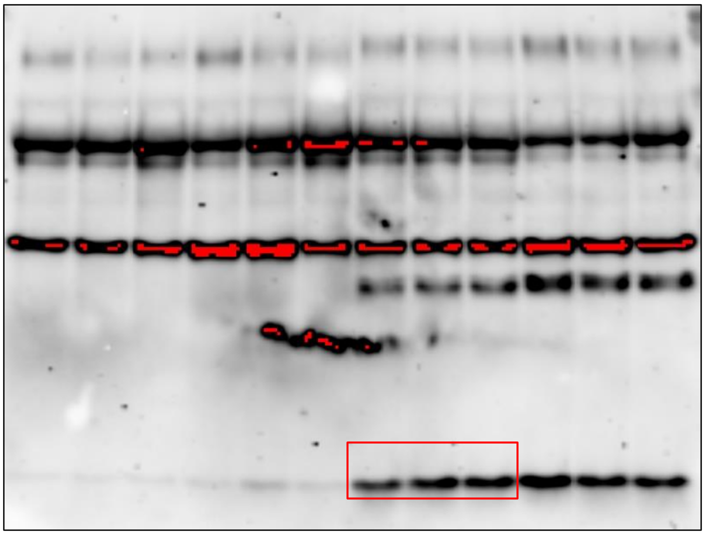

Figure 4G.
GAPDH
Cell Signaling
Catalog #2118
1:25,000

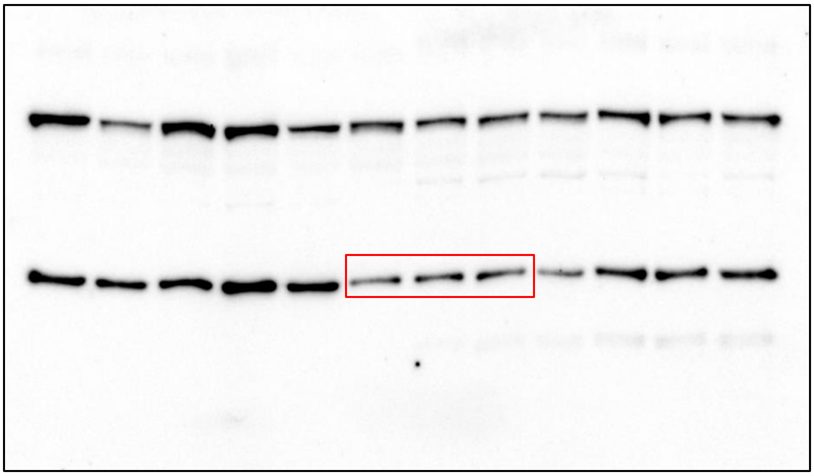

Figure 5D-E.
HM13
Abcam
Catalog #ab247061
1:500

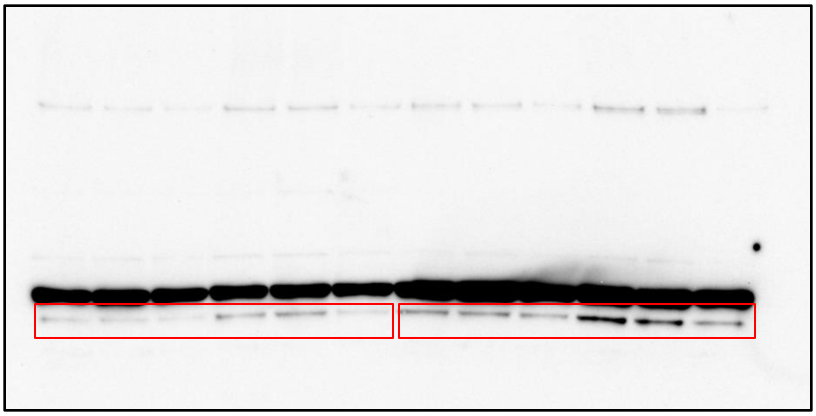

Figure 5D-E.
HSP60
CST
Catalog #12165
1:7500

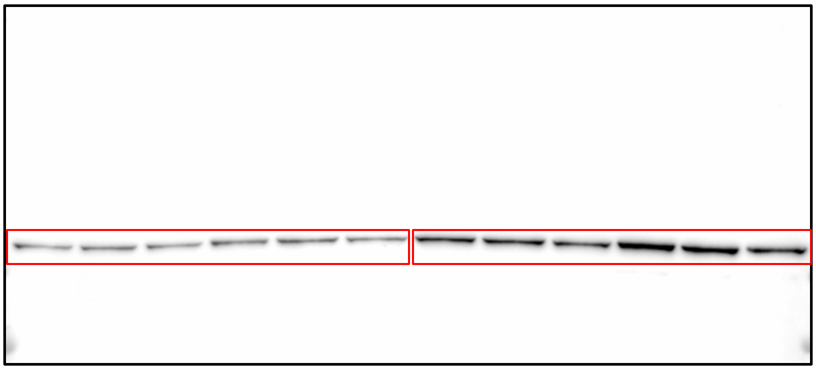

Figure 6A.
BIP
Cell Signaling
Catalog #3177
1:1000 dilution

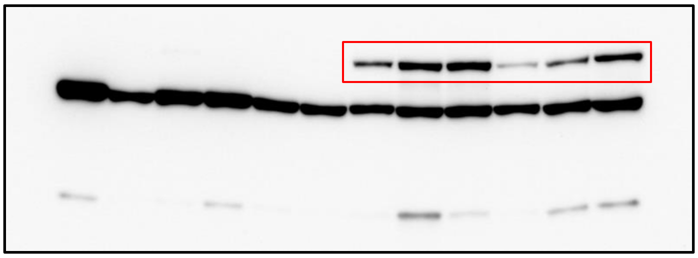

Figure 6A.
PERK
Cell Signaling
Catalog #5683
1:1000 dilution

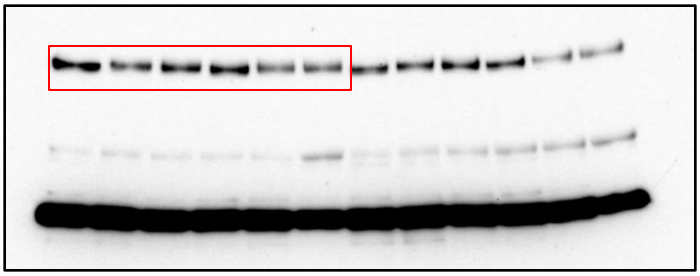

Figure 6A.
GADD34
Invitrogen
Catalog #PA1-139
1:1000 dilution

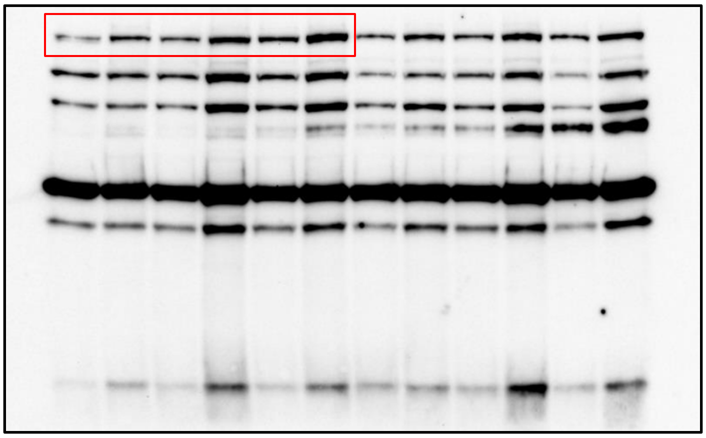

Figure 6A.
ATF-4
Cell Signaling
Catalog #11815
1:1000 dilution

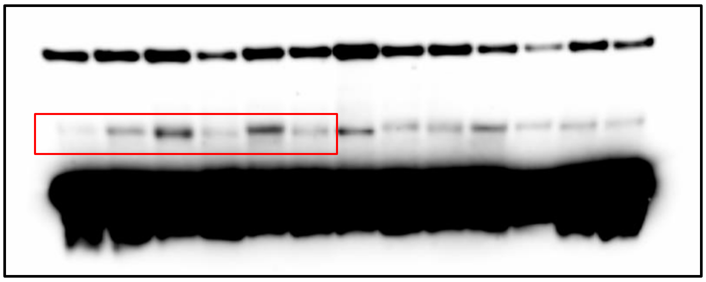

Figure 6A.
p-eIF2a
Cell Signaling
Catalog #3597
1:1000 dilution

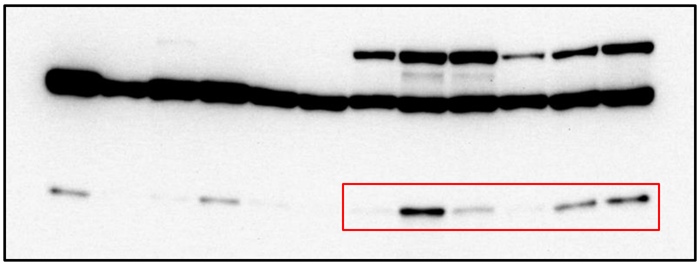

Figure 6A.
IRE-1α
Cell Signaling
Catalog #3294
1:1000 dilution

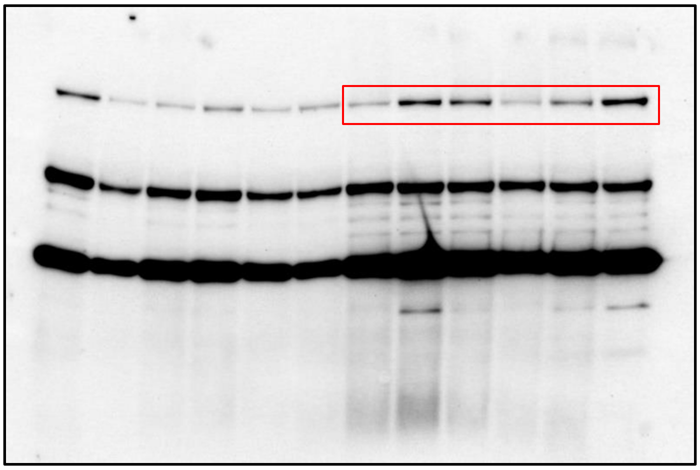

Figure 6A.
XBP-1
Cell Signaling
Catalog #40435
1:1000 dilution

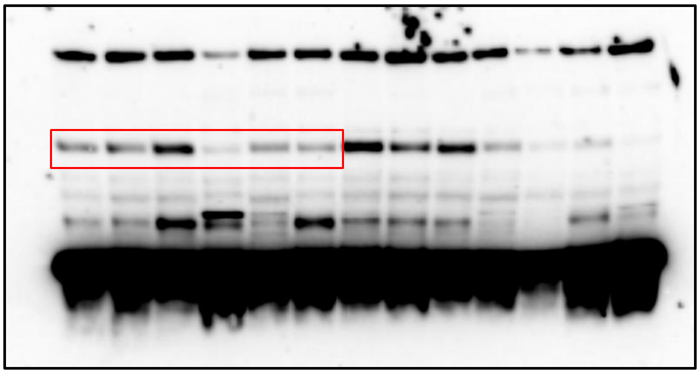

Figure 6A.
p-JNK
Cell Signaling
Catalog #4668
1:1000 dilution

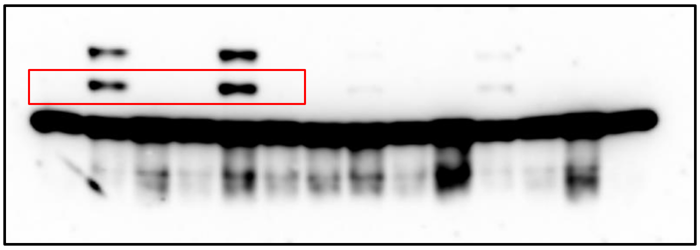

Figure 6A.
ATF-6
Cell Signaling
Catalog #65880
1:1000 dilution

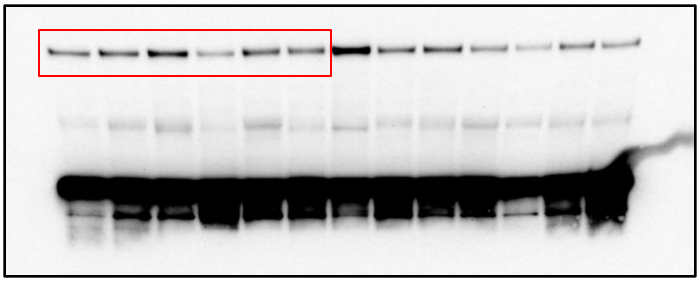

Figure 6A.
PINK1
Cell Signaling
Catalog #6946
1:1000 dilution

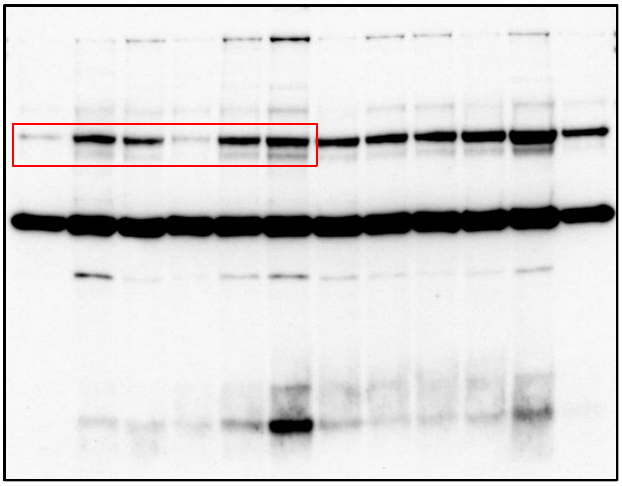

Figure 6A.
NDP52
Abcam
Catalog #68588
1:1000 dilution

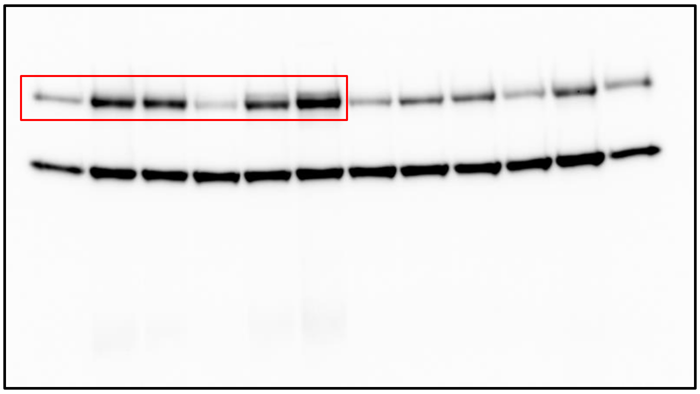

Figure 6A.
LC3
Cell Signaling
Catalog #4108
1:1000

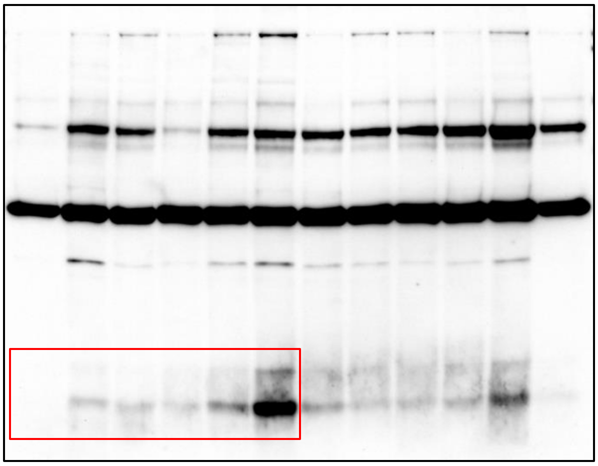

Figure 6A.
CL-PARP
Cell Signaling
Catalog #9532
1:1000

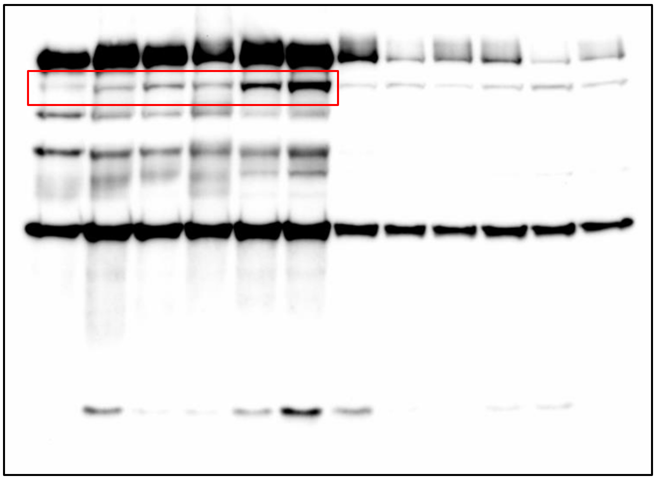

Figure 6A.
Bak
Cell Signaling
Catalog #12105
1:1000

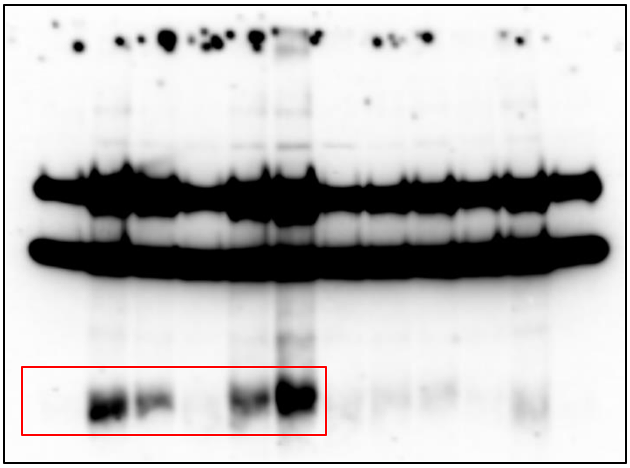

Figure 6A.
HSP60
Cell Signaling
Catalog #12165
1:7500

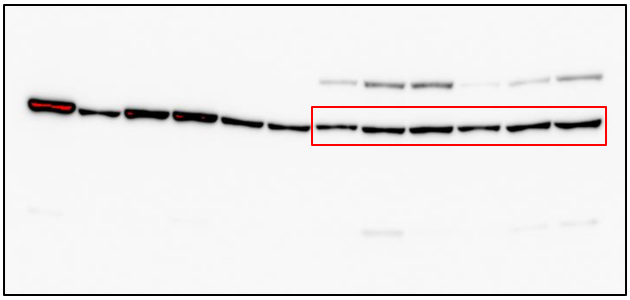

Figure 6A.
GAPDH
Cell Signaling
Catalog #2118
1:25000

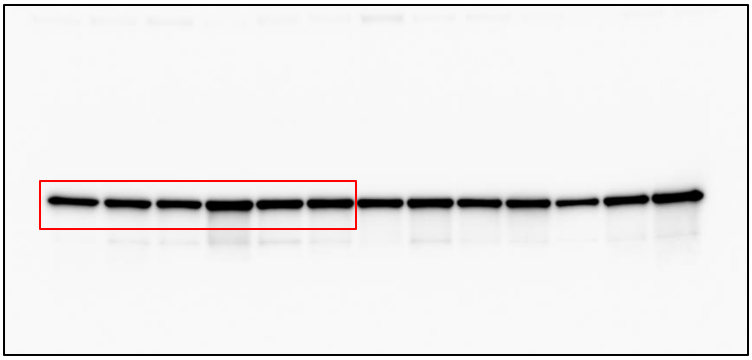

Figure 6B.
PERK
Cell Signaling
Catalog #5683
1:1000

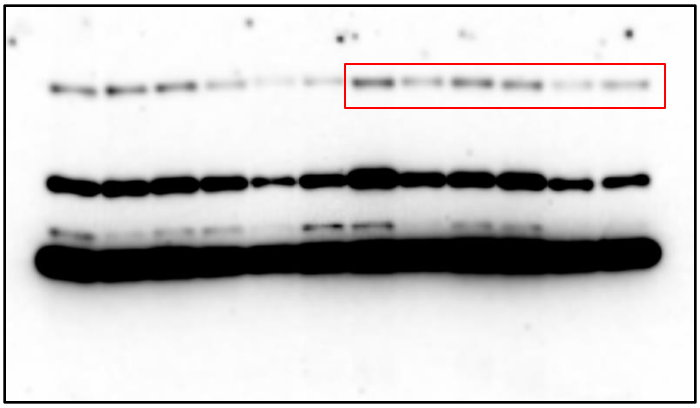

Figure 6B.
GADD34
Invitrogen
Catalog #PA1-139
1:1000

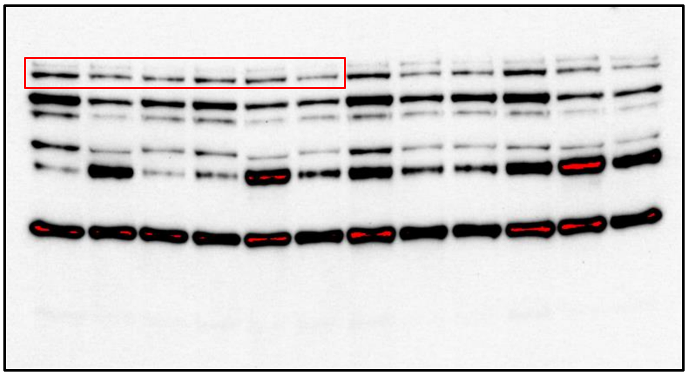

Figure 6B.
IRE-1α
Cell Signaling
Catalog #3294
1:1000

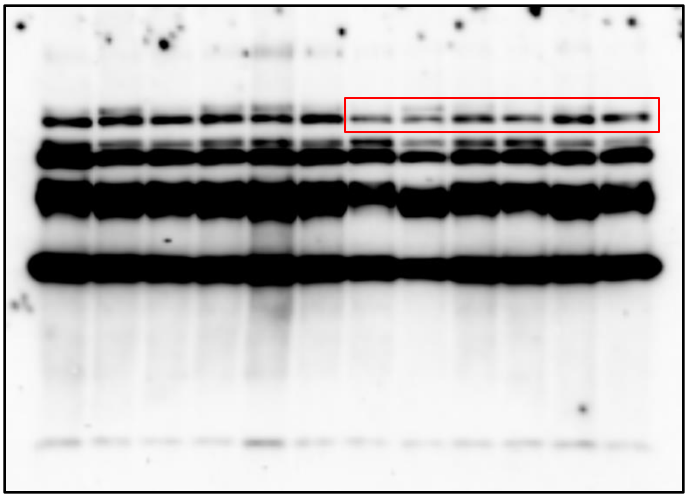

Figure 6B.
XBP1
Cell Signaling
Catalog #40435
1:1000

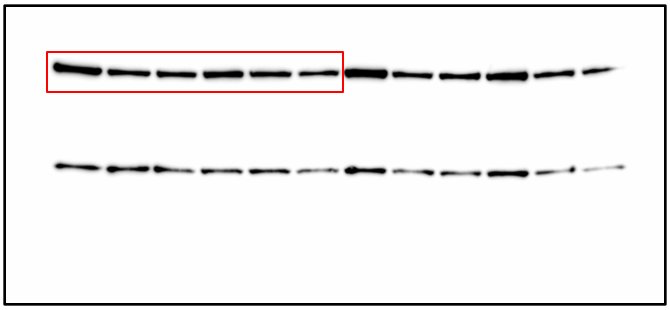

Figure 6B.
ATF-6
Cell Signaling
Catalog #65880
1:1000

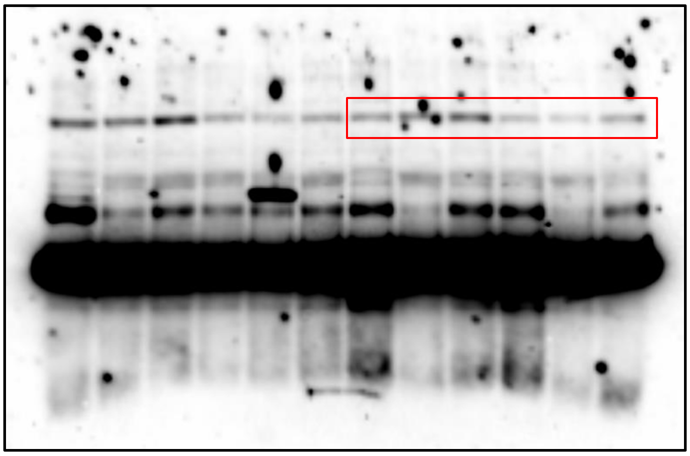

Figure 6B.
PINK1
Cell Signaling
Catalog #6946
1:1000

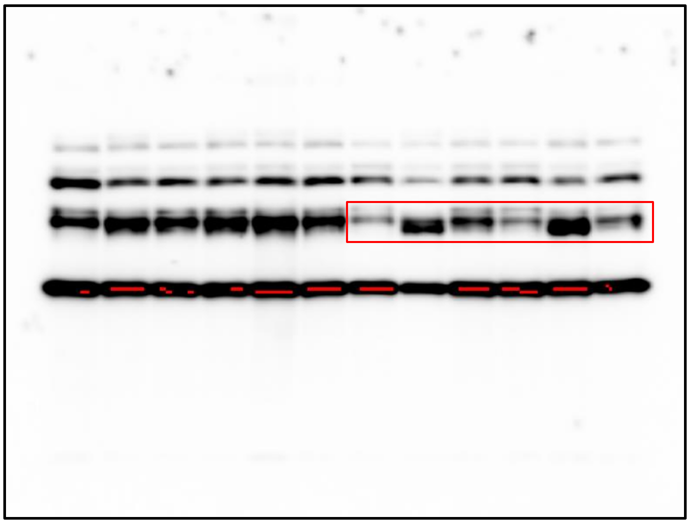

Figure 6B.
NDP52
Abcam
Catalog #68588
1:1000

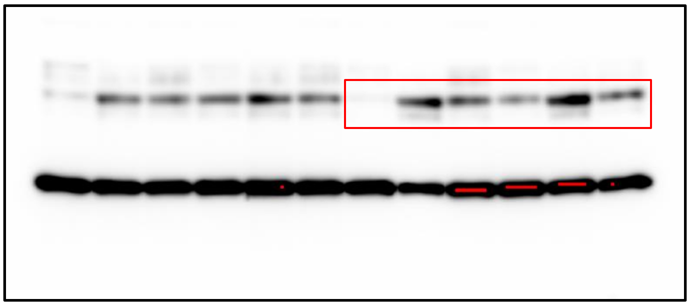

Figure 6B.
LC3
Cell Signaling
Catalog #4108
1:1000

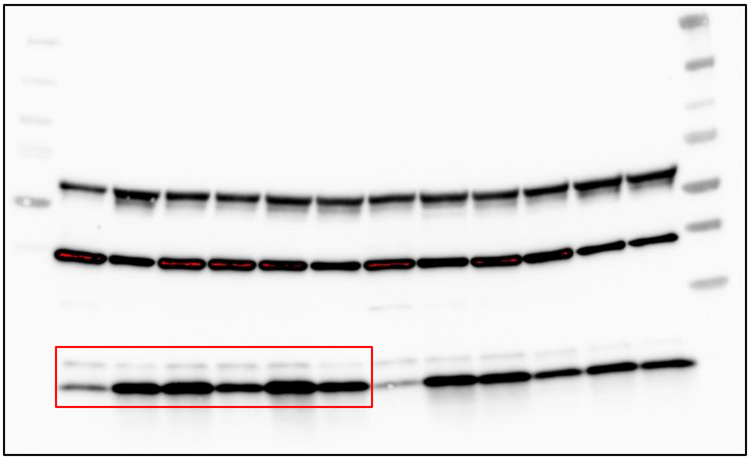

Figure 6B.
CL-PARP
Cell Signaling
Catalog #9532
1:25000

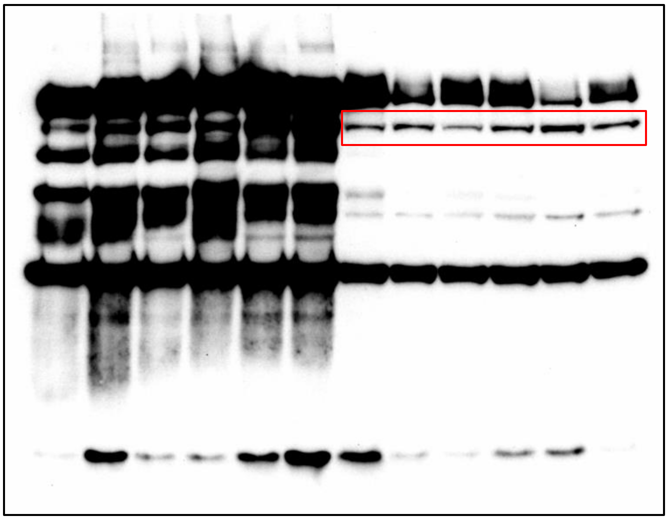

Figure 6B.
GAPDH
Cell Signaling
Catalog #2118
1:25000

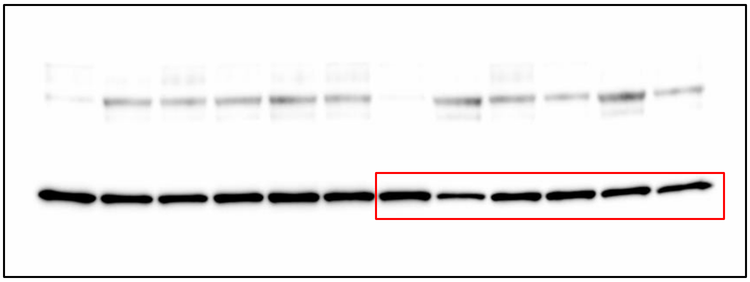

Figure 6D.
Puromycin
DSHB
Catalog #PMY-2A4
1:1000

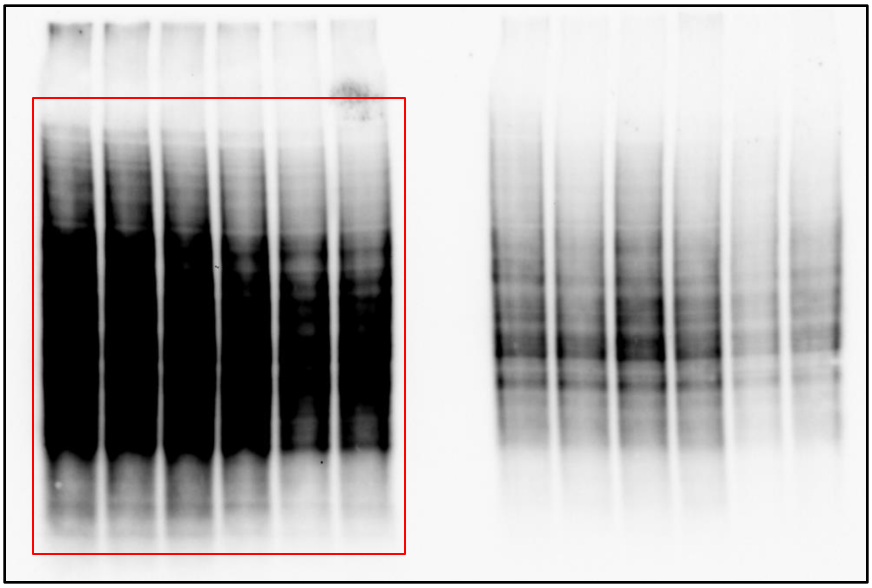

Figure 6D.
GAPDH
Santa Cruz
Catalog #sc-32233
1:30000

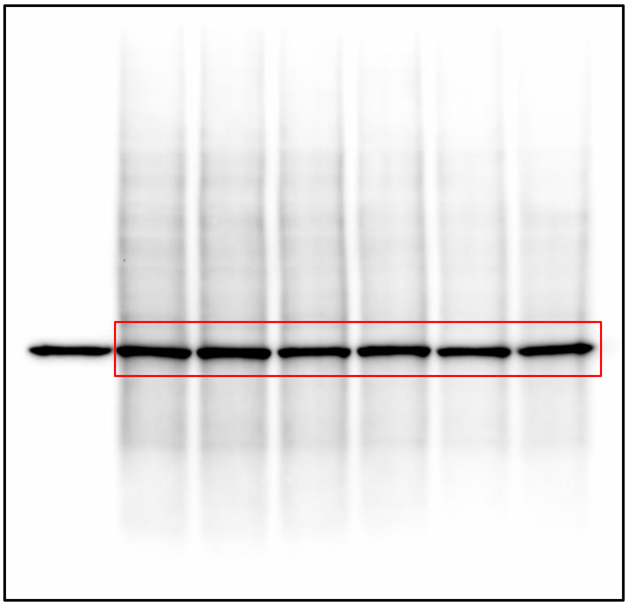

Figure 6E.
Puromycin
DSHB
Catalog #PMY-2A4
1:1000

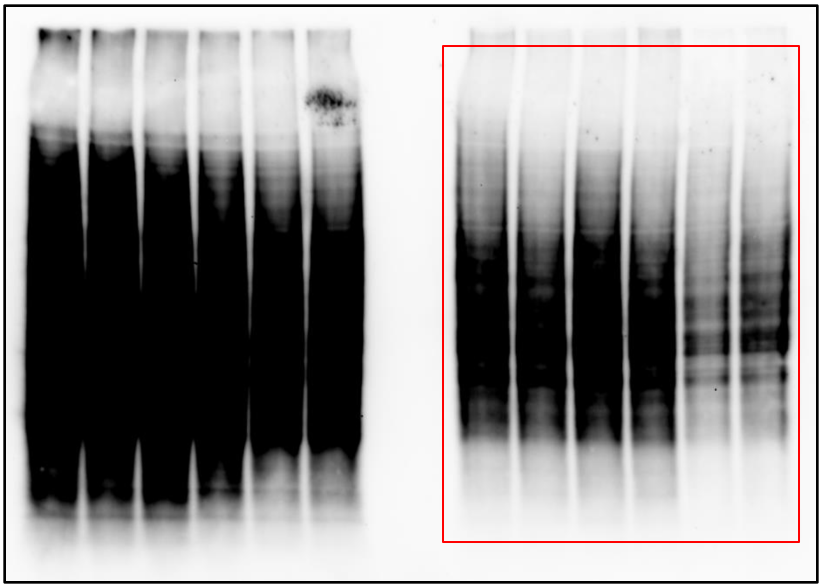

Figure 6E.
GAPDH
Santa Cruz
Catalog #sc-32233
1:30000

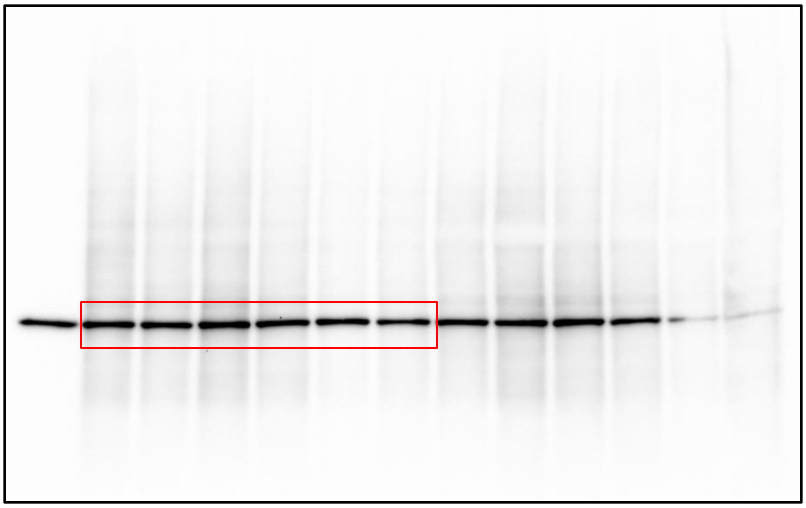

Figure 7C.
BIP
Cell Signaling
Catalog #3177
1:1000

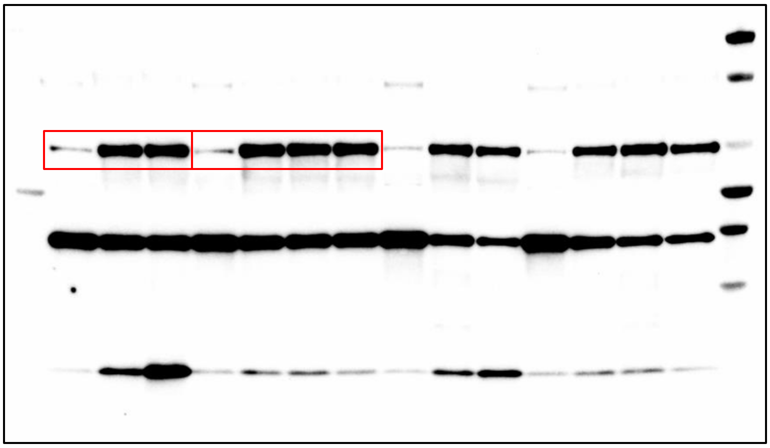

Figure 7C.
PERK
Cell Signaling
Catalog #5683
1:1000

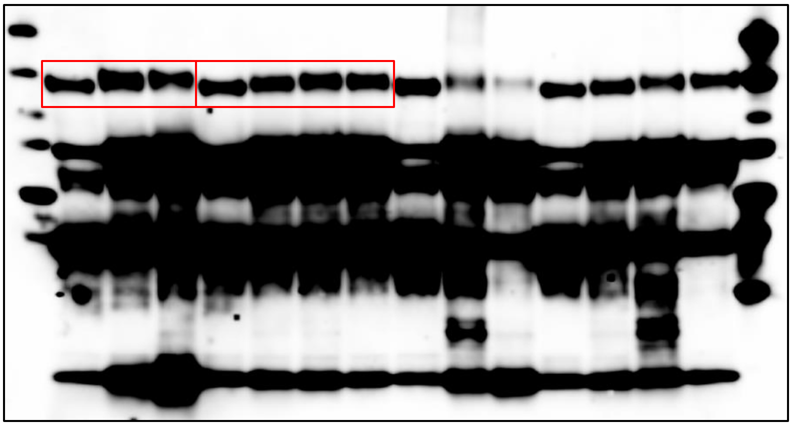

Figure 7C.
GADD34
Invitrogen
Catalog #PA1-139
1:1000

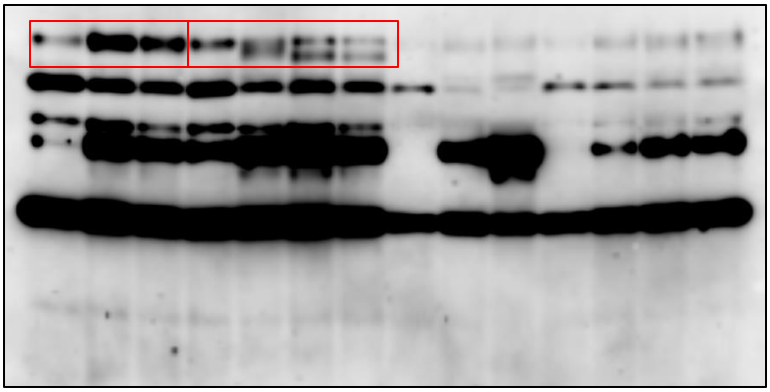

Figure 7C.
p-eIF2a
Cell Signaling
Catalog #3597
1:1000

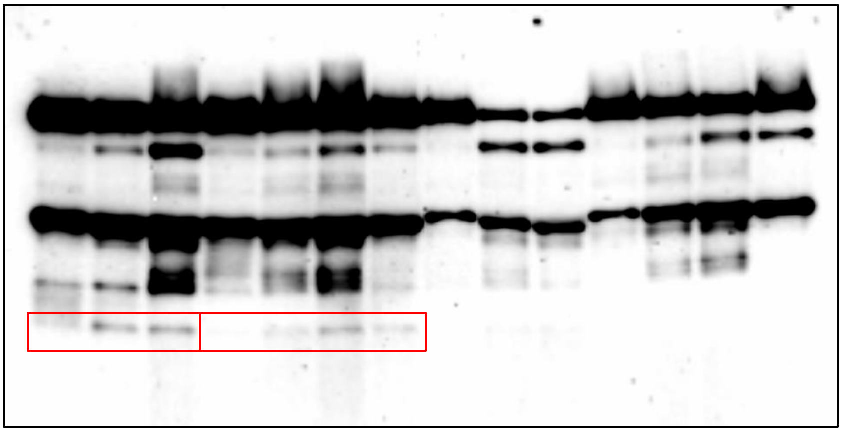

Figure 7C.
IRE-1α
Cell Signaling
Catalog #3294
1:1000

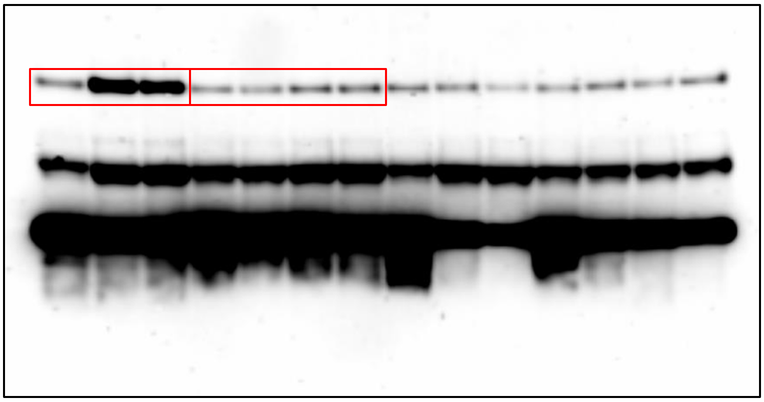

Figure 7C.
XBP-1
Cell Signaling
Catalog #40435
1:1000

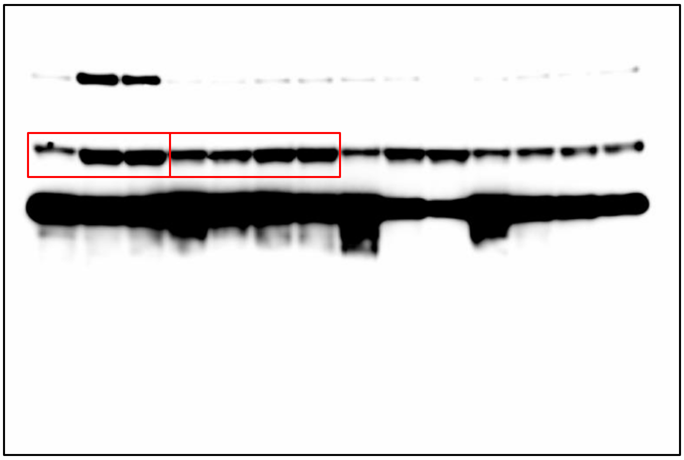

Figure 7C.
p-JNK
Cell Signaling
Catalog #4668
1:1000

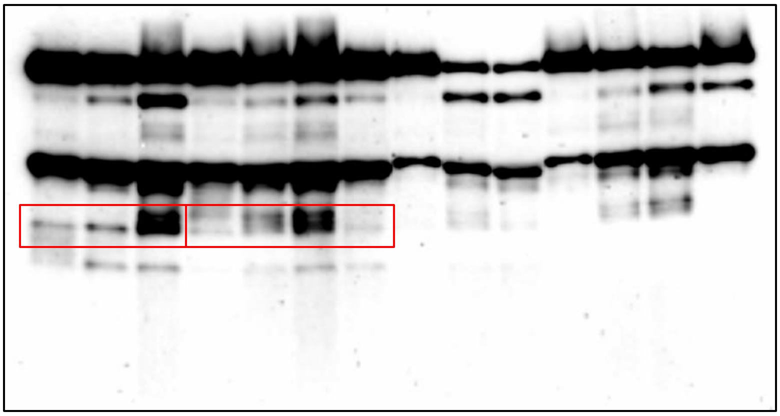

Figure 7C.
ATF-6
Cell Signaling
Catalog #65880
1:1000

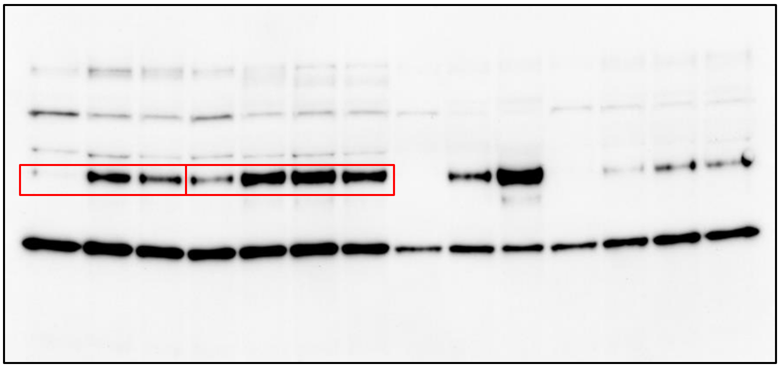

Figure 7C.
LC3
Cell Signaling
Catalog #4108
1:1000

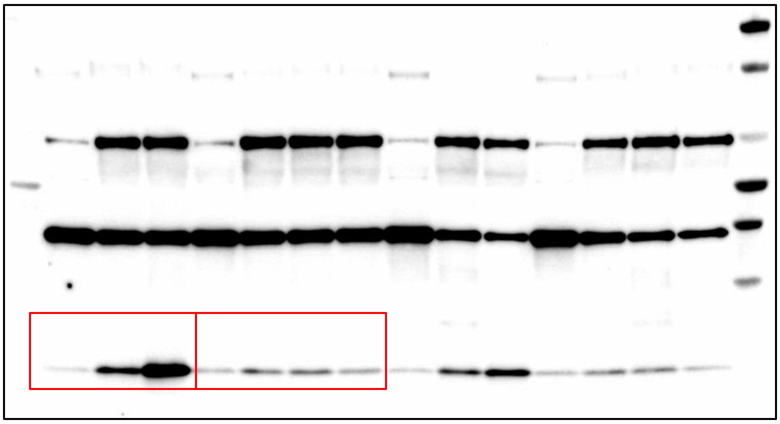

Figure 7C.
CL-PARP
Cell Signaling
Catalog #9532
1:1000

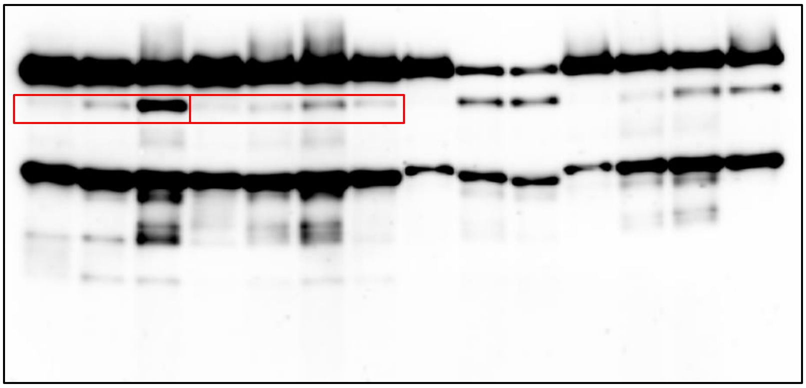

Figure 7C.
GAPDH
Cell Signaling
Catalog #2118
1:25000

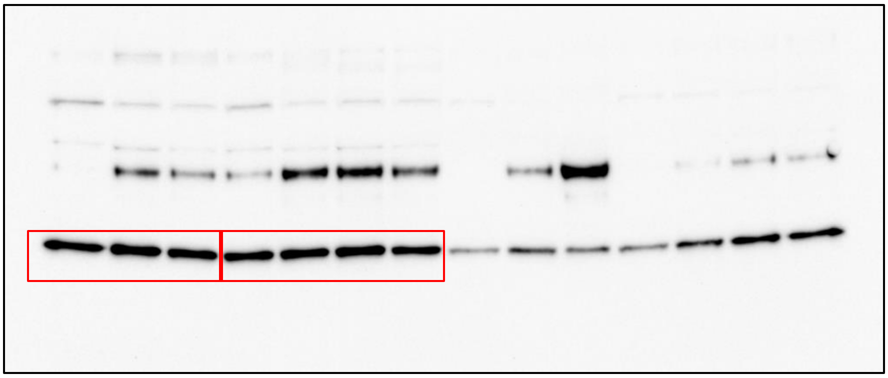

Figure 7D.
PERK
Cell Signaling
Catalog #5683
1:1000

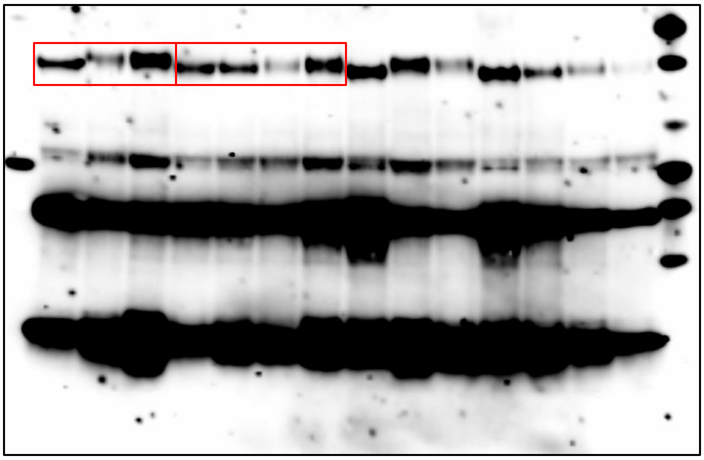

Figure 7D.
GADD34
Invitrogen
Catalog #PA1-139
1:1000

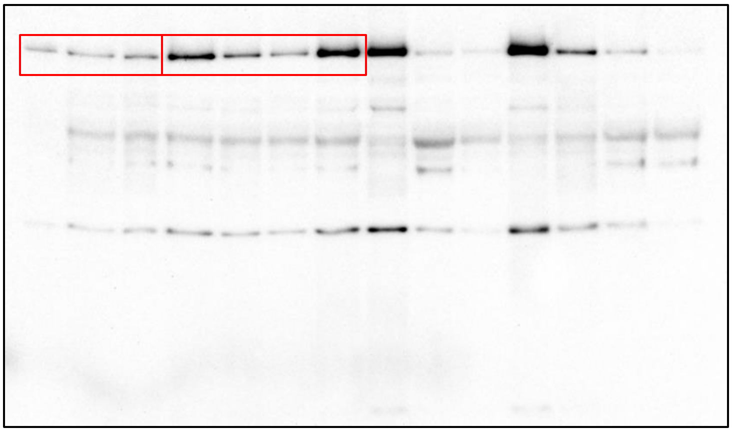

Figure 7D.
IRE-1α
Cell Signaling
Catalog #3294
1:1000

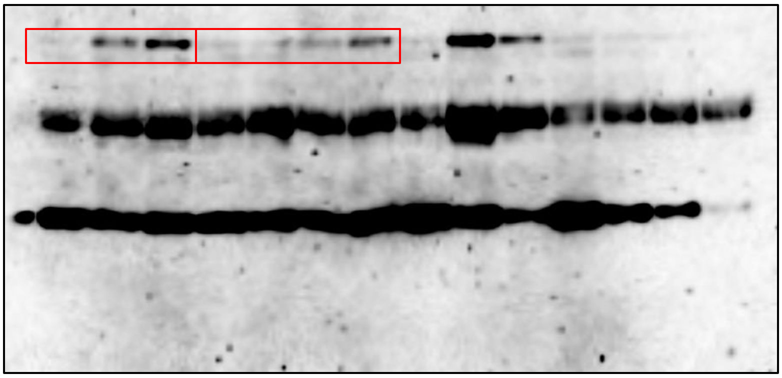

Figure 7D.
XBP-1
Cell Signaling
Catalog #40435
1:1000

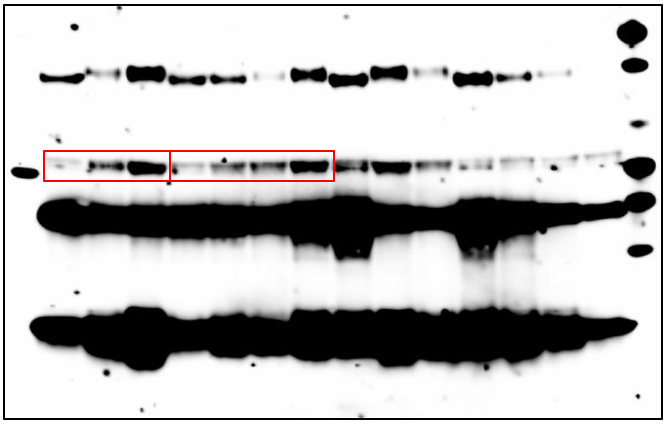

Figure 7D.
ATF-6
Cell Signaling
Catalog #65880
1:1000

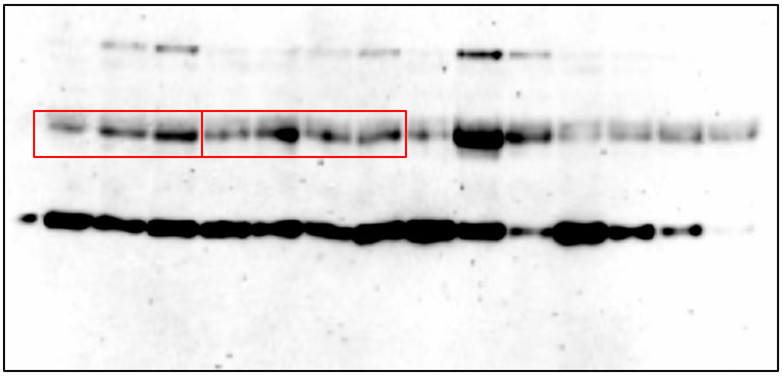

Figure 7D.
LC3
Cell Signaling
Catalog #4108
1:1000

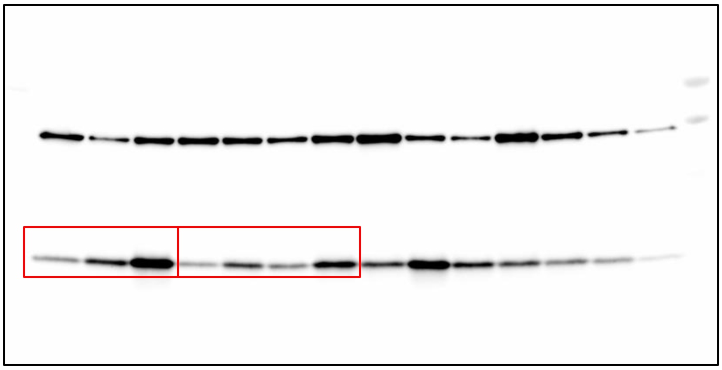

Figure 7D.
CL-PARP
Cell Signaling
Catalog #9532
1:1000

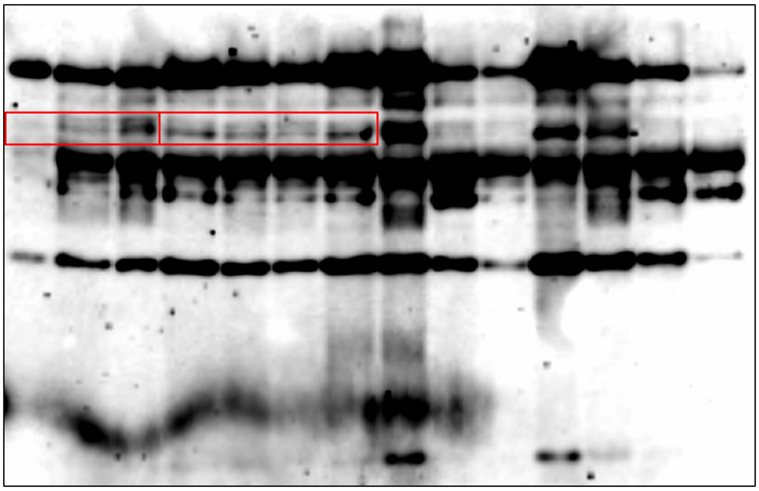

Figure 7D.
GAPDH
Cell Signaling
Catalog #2118
1:25000

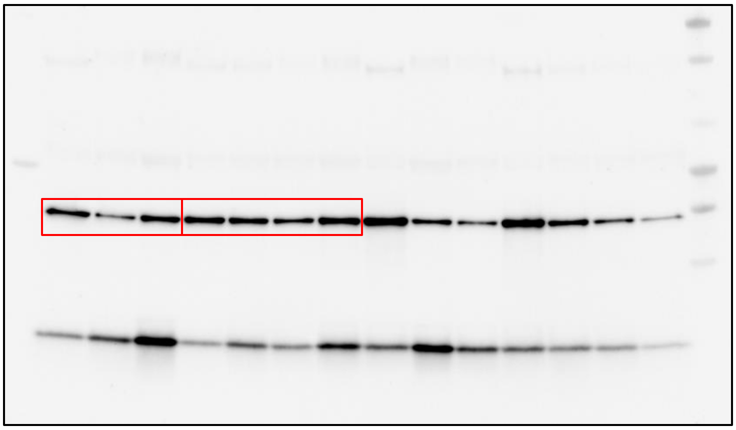

Figure S6.
CHK1
Cell Signaling
Catalog #2360
1:1000

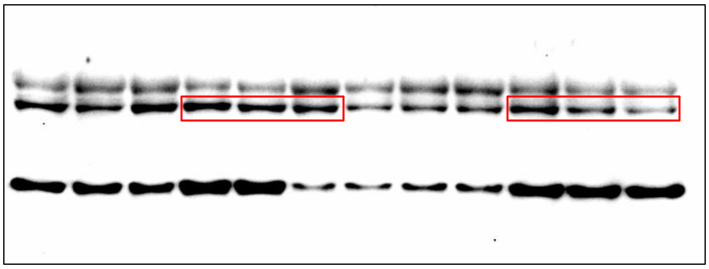

Figure S6.
GAPDH
Cell Signaling
Catalog #2118
1:25000

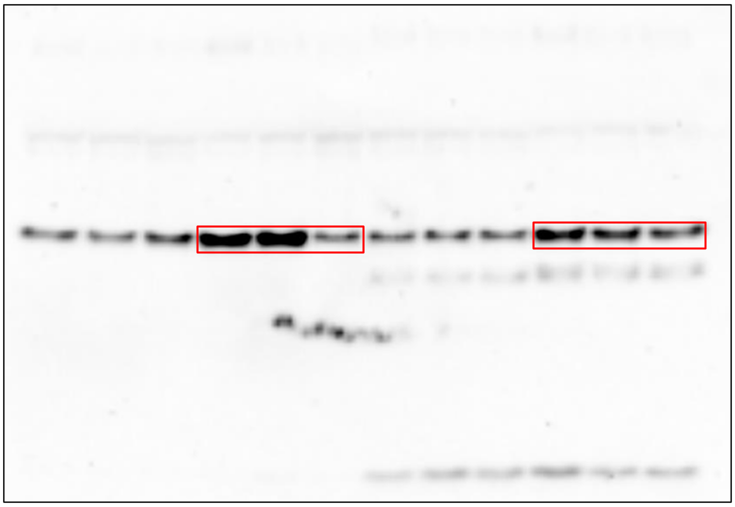

Figure S6.
p-CHK1
Cell Signaling
Catalog #2348
1:1000

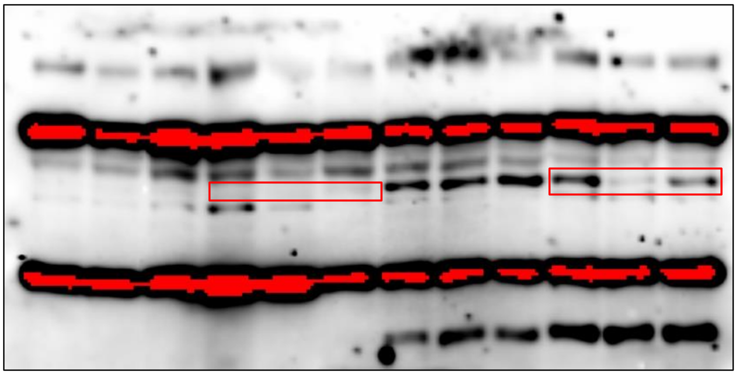

Supplement: Supplementary file 2 — Western blots, Full length, Uncropped, Original blots [file 41419_2026_8542_MOESM2_ESM.pdf]
